# Triethylene Glycol Up-Regulates Virulence-Associated Genes and Proteins in *Streptococcus mutans*

**DOI:** 10.1371/journal.pone.0165760

**Published:** 2016-11-07

**Authors:** Lida Sadeghinejad, Dennis G. Cvitkovitch, Walter L. Siqueira, J. Paul Santerre, Yoav Finer

**Affiliations:** 1 Dental Research Institute, Faculty of Dentistry, University of Toronto, Toronto, Ontario, Canada; 2 Institute of Biomaterials and Biomedical Engineering, University of Toronto, Toronto, Ontario, Canada; 3 Schulich Dentistry and Department of Biochemistry, University of Western Ontario, London, Ontario, Canada; LSU Health Sciences Center School of Dentistry, UNITED STATES

## Abstract

Triethylene glycol dimethacrylate (TEGDMA) is a diluent monomer used pervasively in dental composite resins. Through hydrolytic degradation of the composites in the oral cavity it yields a hydrophilic biodegradation product, triethylene glycol (TEG), which has been shown to promote the growth of *Streptococcus mutans*, a dominant cariogenic bacterium. Previously it was shown that TEG up-regulated *gtfB*, an important gene contributing to polysaccharide synthesis function in biofilms. However, molecular mechanisms related to TEG’s effect on bacterial function remained poorly understood. In the present study, *S*. *mutans* UA159 was incubated with clinically relevant concentrations of TEG at pH 5.5 and 7.0. Quantitative real-time PCR, proteomics analysis, and glucosyltransferase enzyme (GTF) activity measurements were employed to identify the bacterial phenotypic response to TEG. A *S*. *mutans vicK* isogenic mutant (SMΔvicK1) and its associated complemented strain (SMΔvicK1C), an important regulatory gene for biofilm-associated genes, were used to determine if this signaling pathway was involved in modulation of the *S*. *mutans* virulence-associated genes. Extracted proteins from *S*. *mutans* biofilms grown in the presence and absence of TEG were subjected to mass spectrometry for protein identification, characterization and quantification. TEG up-regulated *gtfB/C*, *gbpB*, *comC*, *comD* and *comE* more significantly in biofilms at cariogenic pH (5.5) and defined concentrations. Differential response of the *vicK* knock-out (SMΔvicK1) and complemented strains (SMΔvicK1C) implicated this signalling pathway in TEG-modulated cellular responses. TEG resulted in increased GTF enzyme activity, responsible for synthesizing insoluble glucans involved in the formation of cariogenic biofilms. As well, TEG increased protein abundance related to biofilm formation, carbohydrate transport, acid tolerance, and stress-response. Proteomics data was consistent with gene expression findings for the selected genes. These findings demonstrate a mechanistic pathway by which TEG derived from commercial resin materials in the oral cavity promote *S*. *mutans* pathogenicity, which is typically associated with secondary caries.

## Introduction

Over the past few decades, resin composites have been widely used as dental restorative materials. This is due to their superior aesthetics, excellent adhesive strength to dentin and enamel, minimal intervention approaches to restore the posterior teeth and concern related to possible adverse effects of mercury released from dental amalgam [1]. Since their development in the 1960s, the basic properties of resin composites such as mechanical, physical and bonding properties have been remarkably improved. However, a number of clinical studies have reported higher failure rates, increased frequency of replacement and shorter longevity for composite restorations compared to amalgams [1–6]. One of the main reasons for resin composite restoration failure is secondary or recurrent caries [1–3, 5, 7–10]. Furthermore, the result of two most recent systematic reviews suggested that resin composite restorations in posterior teeth have less longevity and a higher number of secondary caries when compared to amalgam restorations [11, 12]. Based on one of these systematic reviews, the incidence of secondary caries around amalgams varied between 0% and 4.9%, but composite restorations tend to exhibit markedly more secondary caries with incidences varying between 0% and 12.7% [12]. These findings necessitate more fundamental research to unravel all underlying causes promoting secondary caries, as premature replacement of resin composite restorations due to secondary caries imposes a tremendous burden on health care expenditure [13]. The total cost of dental restoration is approximately $46 billion/year in the U.S.A and 70% of this cost is related to the replacement of failed restorations [14, 15]. Failed composite restorations are responsible for more than half of all dental restorations [16].

The polymeric matrix of commonly used composite resin restorations typically contain a viscous dominant hydrophobic monomer, bis-phenyl glycidyl dimethacrylate (BisGMA), as well as dilutive hydrophilic monomer such as triethylene glycol dimethacrylate (TEGDMA) [17]. While TEGDMA has many advantages such as rapid conversion and setting in the oral cavity and ease of manipulation [17], it is highly susceptible to hydrolytic biodegradation, catalyzed by human and bacterial esterases [18, 19]. The reason for this susceptibility is the presence of an unprotected ester linkage within its structure. The degradation of TEGDMA results in the production of a biodegradation by-product called tri-ethylene-glycol (TEG) [20–24].

The degradation process contributes to the deterioration of the interface and ingress of cariogenic microorganisms such as *Streptococcus mutans* that can potentially result in the development of secondary caries [25]. *S*. *mutans* is one of the leading species associated with human dental caries and is considered to be the most cariogenic of all the oral streptococci [26]. A previous study by the authors reported that TEG can up-regulate the expression of a virulence gene, *gtfB*, which encodes a glucosyltransfrerase (GTF) enzyme in *S*. *mutans* NG8. TEG up-regulated *gtfB* in both planktonic and biofilm cells and at both cariogenic (5.5) and neutral (7.0) pH [27]. However, the downstream impact of this upregulation on molecular pathways that control metabolism, acid production, potential for biofilm formation, and other cell functions remained unknown. Despite the limited time frame in which TEGDMA is being released from new resin composite restorations [23], most studies to date have focused on the interactions of this monomer with biological systems in the oral cavity, investigating the cytotoxic, genotoxic and estrogenic effects on both mammalian and bacterial cells. Yet, there are only very limited studies that examined the biological effects of the degradation by-product from this monomer, TEG, which is released over a long term, throughout the life of the restoration [23].

In the present study, we used a well-characterized reference strain of *S*. *mutans*, UA159, with its fully annotated genome sequence available to enable a more thorough investigation of individual gene expression analysis and the synthesis of its proteins [28]. *S*. *mutans* biofilms were exposed to clinically-relevant concentrations of TEG (0.001–1.0 mM) [29] at cariogenic and neutral pH.

The objectives of the present study were: *i)* to elucidate the effects of TEG on the expression of known *S*. *mutans* virulence genes, *i*.*e*. *gtfB*, *gtfC*, *gbpB*, *comC*, *comD*, *comE* and *atpH* using qRT-PCR; *ii)* to identify potential molecular pathway(s) involved in those effects; *iii)* to quantify global protein synthesis and identify other molecular pathways that were affected by interaction of TEG with the bacteria using proteomics, and finally; *iv)* to measure the effect of TEG on *S*. *mutans* GTF enzyme activity, associated with biofilm formation, to confirm that the effect of TEG on *gtfB* and *gtfC* expression could result in increased production of water-insoluble exopolysaccharide glucan, a known virulence factor in *S*. *mutans* [30]. The seven selected genes in this study have unique virulence properties in *S*. *mutans* and have been linked to its cariogenicity by previous preclinical and clinical studies [31–38].

*S*. *mutans* isogenic mutants deleted in a key regulatory gene, *vicK*, (SMΔvicK1) and its associated complemented strain (SMΔvicK1C) were used to identify the molecular pathway(s) involved in the TEG-mediated cellular effect. *VicRK* is one of the 14 two-component signal transduction systems (TCSTSs) in *S*. *mutans* [28] that regulates the *comCDE* system, *gtfB*, *gtfC*, *gbpB* expression and acid tolerance in *S*. *mutans* [39–43]. TCSTSs in *S*. *mutans* respond to environmental stimuli by transmitting signals from a membrane-associated histidine kinase (HK) to an intracellular response regulator (RR) protein via transphosphorylation, which in turn, regulates the transcription of its target genes [44]. Therefore, the current study investigated whether the effects of TEG on *S*. *mutans* gene expression are regulated by the *vicRK* system.

To the best of the authors’ knowledge, the current investigation provides the first proteomics analysis of cariogenic *S*. *mutans* UA159 after exposure to the dental composite degradation product TEG. The implications of the work are significant given that TEGDMA is pervasively used in oral restorations around the world.

## Materials & Methods

### Bacterial Strain and Growth Conditions

The *S*. *mutans* UA159 wild-type strain was obtained from Dr. Arnold Bleiweis (University of Florida) and stored in 20% glycerol at -80°C. To construct the *vicK*-deficient mutant (SMΔvicK1), a PCR-ligation mutagenesis strategy was used as previously described [45, 46]. The *vicK* complemented strain (SMΔvicK1C) was made using pIB166 to replace the *vicK* gene with an *erm* resistant gene cassette by a double crossover recombination event. Then a constitutive vector pIB166 harboring the full length *vicK* gene between *BamH*I and *Xho*I cutting sites was transformed into a *vicK*-deletion mutant to generate the complemented strain. The primers used for the *vicK* deletion and complementation constructs are listed in Table 1 (Operon, AL, USA). The *S*. *mutans* wild-type strain was sub-cultured on a Todd-Hewitt agar plate supplemented by 0.3% yeast extract (THYE) (BBL; Becton Dickenson, Cockeysville, MD, USA), whereas the *vicK* mutant was maintained on a THYE agar containing 10 μg/mL of erythromycin [45]. All *S*. *mutans* overnight cultures were routinely grown in THYE broth at 37°C in a 5% CO2−95% air mixtures.

**Table 1:** Primers used for construction of *vicK* knock-out and complemented strains.

Primer use and name	Nucleotide Sequence
**Primers for *vicK* deletion:**	
**VicK-P1**	GTTACCAGAACTAGACGGTCTTGAAG
**VicK-P2**	GG^CGCGCCTTAGTCATATGATTTCATGTAATAACCAAC
**VicK-P3**	GGCCGG^CCATGACAGAAACAGGTTTTAGATAC
**VicK-P4**	AAACTCCAAGGGTAGATACCTG
**Erm cst-F**	GG^CGCGCCCCGGGCCCAAAATTTGTTTGAT
**Erm cst-B**	GGCCGG^CCAGTCGGCAGCGACTCATAGAAT
**Primers for VicK complementation:**	
**VicK-F**	5' ggattcATGACTAATGTGTTTGAATCAAG 3'
**VicK-R**	5' ctcgagTCATGATTCGTCTTCATCTTC 3'

### Preparation of Planktonic Cells

Overnight cultures of *S*. *mutans* UA159 were diluted (1/10) in TYEG medium containing tryptone (10 g/L), yeast extract (5 g/L) and glucose (5 mM) buffered either at pH 5.5 with 100 mM MES (2-(N-Morpholino)-ethanesulfonic acid, Sigma-Aldrich, St. Louis, MO, USA) or at pH 7.0 with 100 mM MOPS (3-(N-Morpholino) propanesulfonic acid, Bioshop, Burlington, ON, Canada) to control pH, supplemented with 0.1% glucose and 500 μL of the appropriate amount of sterile filtered TEG (99.9% pure, Sigma-Aldrich) yielding final concentrations of 0, 0.001, 0.01, 0.1 mM TEG in the solutions [29]. The culture tubes were incubated for 18 hours (37°C, 5% CO_2_). The pH of sample cultures was verified (H135 minilab^TM^ pro, HACH, Germany). Bacterial cell density was monitored using a UV spectrophotometer (Ultraspec 3000, Biotech) at 600 nm in order to ensure that the cultures were harvested once they reached mid-logarithmic phase (optical density or OD = 0.4). This was followed by centrifugation at 2300 g for 10 minutes (4°C). The supernatants were discarded and the pellets were snap frozen in liquid nitrogen and stored at -80°C until required for RNA isolation.

### Preparation of Biofilm Cells

Overnight cultures of *S*. *mutans* UA159 were diluted (1:60) in TYEG medium and added to six-well polystyrene microtiter plates (Fisher Scientific). Each well containing 3 mL of ¼ strength TYEG medium buffered to pH 5.5 or 7.0 and 50 μL of overnight culture were supplemented with 0.1% glucose and 500 μL of TEG stock solutions as described for planktonic conditions above. Cells were then incubated for 18 hours (37°C, 5% CO_**2**_**)** after which the pH of sample cultures was measured. Then, the liquid contents were removed and 3 mL of phosphate-buffered saline (PBS: 8 g NaCl, 0.2 g KCl, 1.44 g Na2HPO4 and 0.24 g KH2PO4 dissolved in distilled water to make a 1 L solution, pH 7.4) was slowly added to each well and gently stirred to remove loosely attached cells, leaving only adhered biofilm cells. The remaining PBS was then removed and replaced with 1 mL of fresh PBS. Biofilm cells from each well were scraped and the resulting cell suspensions were transferred to 50 mL tubes and centrifuged at 2300 g for 10 minutes (4°C). The supernatants of each sample were then removed/discarded and the pellets were snap frozen in liquid nitrogen and stored at -80°C until required for RNA isolation.

### Gene Expression Analysis Using qRT-PCR

Total RNA was isolated by disruption of *S*. *mutans* UA159 cells (Thermo Savant, Fast-Prep FP 101), followed by DNase treatment of the RNA samples and cDNA synthesis as described before [27]. QRT-PCR was used to quantify the relative gene expression of selected genes: *gtfB*, *gtfC*, *gbpB*, *comC*, *comD*, *comE* and *atpH* as previously described [27]. The primers used are presented in Table 2 (Operon, AL, USA). Quantitative gene expression data were then normalized to the 16S rRNA, a well-established housekeeping gene [47]. The level of 16S rRNA message was not affected by TEG within the concentration range studied (data not shown). QRT-PCR gene expression analysis was also employed to investigate the involvement of the *vicRK* system in regulation of the *S*. *mutans* virulence genes of interest in the presence TEG. Using *vicK* knock-out (SMΔvicK1) and complemented (SMΔvicK1C) strains, gene expression analysis was repeated for three representative genes, *gtfB* (biofilm formation), *comD* (quorum sensing) and *atpH* (acid tolerance), encompassing different functional roles within the virulence character. For statistical analysis, one-way analysis of variance (ANOVA) and Tukey *post hoc* analyses were performed to determine changes in gene expression between different concentrations of TEG and the no-TEG control within each growth mode (p<0.05). Two-way ANOVA and Tukey *post-hoc* analyses were conducted to validate differences in gene expression between growth modes (biofilm *vs*. planktonic) at the same concentration (p<0.05). A 2-fold or greater change in gene expression (up-regulation) and ≤ 0.5-fold (down-regulation) with a *P*-value cut-off of < 0.05 were considered physiologically significant [48]. All qRT-PCR reactions were run in triplicate for each experimental condition and the experiments were reproduced four separate times using four different overnight cultures of *S*. *mutans* UA159.

**Table 2:** Nucleotide sequence of primers used for qRT-PCR.

Gene	Description	Primer Sequence (5'-3')	
		Forward	Reverse
*gtfB*	GTF I, glucan production	ACACTTTCGGGTGGCTTG	GCTTAGATGTCACTTCGGTTG
*gtfC*	GTF II, glucan production	CCAAAATGGTATTATGGCTGTCG	GAGTCTCTATCAAAGTAACGCAGT
*gbpB*	Glucan binding protein	AGCAACAGAAGCACAACCATCAG	CCACCATTACCCCAGTAGTTTCC
*comC*	Competence-stimulating peptide	GACTTTAAAGAAATTAAGACTG	AAGCTTGTGTAAAACTTCTGT
*comD*	Two-component regulatory system	CTCTGATTGACCATTCTTCTGG	CATTCTGAGTTTATGCCCCTC
*comE*	Two-component regulatory system	CCTGAAAAGGGCAATCACCAG	GGGGCATAAACTCAGAATGTGTCG
*atpH*	Acid tolerance	ACCATACATTTCAGGCTG	TTTTAGCACTTGGGATTG
*16Sr*RNA	Normalizing internal standard	CTTACCAGGTCTTGACATCCCG	ACCCAACATCTCACGACACGAG

### Sample Preparation for Mass Spectrometry

Biofilms from both experimental (0.01, 0.1 and 1.0 mM) and control (no TEG) groups were washed twice in cold PBS and re-suspended in 1 mL of PBS buffer. The cells were disrupted using a homogenizer (Thermo Savant, FastPrep FP 101) for 45 minute and then centrifuged at 15700 x g for 1 minute. Supernatant were carefully removed, separated in aliquots of 50 μL and stored at -80°C. The total protein concentration in each sample was assessed by the micro bicinchoninic acid (Micro BCA) assay [49]. Equal amounts of protein (20 μg) from both experimental and control groups were dried by a rotary evaporator, denatured and reduced for 2 hours by the addition of 200 μL of 4 M urea, 10 mM dithiothreitol (DTT), and 50 mM NH_4_HCO_3_, pH 7.8. After four-fold dilution with 50 mM NH_4_HCO_3_, pH 7.8, tryptic digestion was carried out for 18 h at 37°C, following the addition of 2% (w/w) sequencing-grade trypsin (Promega, Madison, WI, USA).

### Liquid Chromatography Electrospray Ionization Tandem Mass Spectrometry (LC-ESI-MS/MS) and Relative Proteome Quantitation

Peptide separation and mass spectrometric analyses were carried out as described previously [49]. The MS/MS spectra were analysed against the streptococcal protein database (Swiss Prot and TrEMBL, Swiss Institute of Bioinformatics, Geneva, Switzerland, http://ca.expasy.org/sprot/) using SEQUEST algorithm in Proteome Discoverer 1.3 software (Thermo Scientific, San Jose, CA, USA) [49].

For quantitative proteome analysis, three MS raw files from each group (control and experimental groups) were analyzed using SIEVE software (Version 2.0 Thermo Scientific, San Jose, CA, USA). For the alignment step, a single MS raw file belonging to the control group (no TEG) was selected as the reference file and all of the other files were adjusted to generate the best correlation to this reference file. After alignment, the feature detection and integration (or framing) process was performed using the MS level data. For statistical analyses of protein abundance, peak integrations were summarized into protein-level annotation in SIEVE using a weighted average of intensities for the LC-ESIMS/MS for each protein. In addition, a statistical model based on an ANOVA framework with Tukey’s *post hoc* test was carried out. All study groups were run 3 separate times to increase coverage of the samples and identify more proteins, with 4 independent samples (using 4 independent *S*. *mutans* cultures) in each group. Relative abundance of an individual protein from different TEG concentration groups was considered significantly different from the control group (no TEG) when the values observed were < 0.5 for decreased abundance and >1.5 for increased abundance with a *P*-value cut-off of < 0.05 [49, 50].

### GTF Enzyme Activity Assay

GTF enzyme activity, related to the cleavage of sucrose into fructose and glucose and then added to the growing exopolysaccharides, was measured by determining the rate that [^14^C]-sucrose was converted to glucan polymers, [51]. Briefly, *S*. *mutans* UA159 biofilms were grown in a 6-well polystyrene microtiter plate containing ¼ TYEG medium buffered to pH 5.5. Defined amounts of TEG were added to the medium to yield the target final concentrations (0.00, 0.01, 0.1 and 1.0 mM). Overnight cultures were added to the mixture and incubated for 18 hours. Bacterial cells were collected, transferred to 50 mL tubes and pelleted by centrifugation at 2300 X g at 4°C for 10 minutes. The supernatant was then removed/discarded and the pellets were washed twice in cold PBS and re-suspended in 1 mL of PBS buffer. The cells were disrupted using a homogenizer (Thermo Savant, FastPrep FP 101) for 45 min and then centrifuged at 15,700 X g for 1 minute. Different aliquots of the supernatant were carefully removed and stored separately at -80°C. Total protein concentration in each sample was assessed by the micro bicinchoninic acid (Micro BCA) assay. Fifteen μg of protein from both the experimental and control groups was added to 0.2 M potassium phosphate buffer (pH 6.8) for a total volume of 20 μL, this solution was mixed with 20 μL of ^14^C-radiolabelled reaction buffer containing 0.2 M KPO_4_ (pH6.8), 20 mM sucrose, and 10 μL/mL ^14^C-sucrose (24.4 GBq/mmol; Amersham). The mixture was incubated at 37°C for 60 minutes after which the reaction mixtures were adsorbed onto 25mm filters (0.22 μm GVWP; Millipore). The samples were then air dried for 20 minutes and washed three times with 2 mL distilled water to remove the water-soluble glucan and serve only as water-insoluble glucan samples. The water-insoluble glucan, mainly synthesized by GtfB and GtfC enzymes. The samples were then placed in 5 mL scintillation fluid (ScintiSafe Econo 2 Cocktail; Fisher) and synthesized [^14^C]-glucan was measured using a liquid scintillation counter (Beckman LS6500). One way ANOVA and Tukey *post hoc* analyses were used to determine significance in GTF activity.

## Results

### Effects of TEG on *S*. *mutans* UA159 Gene Expression at pH 5.5 and 7.0

**Fig 1:**
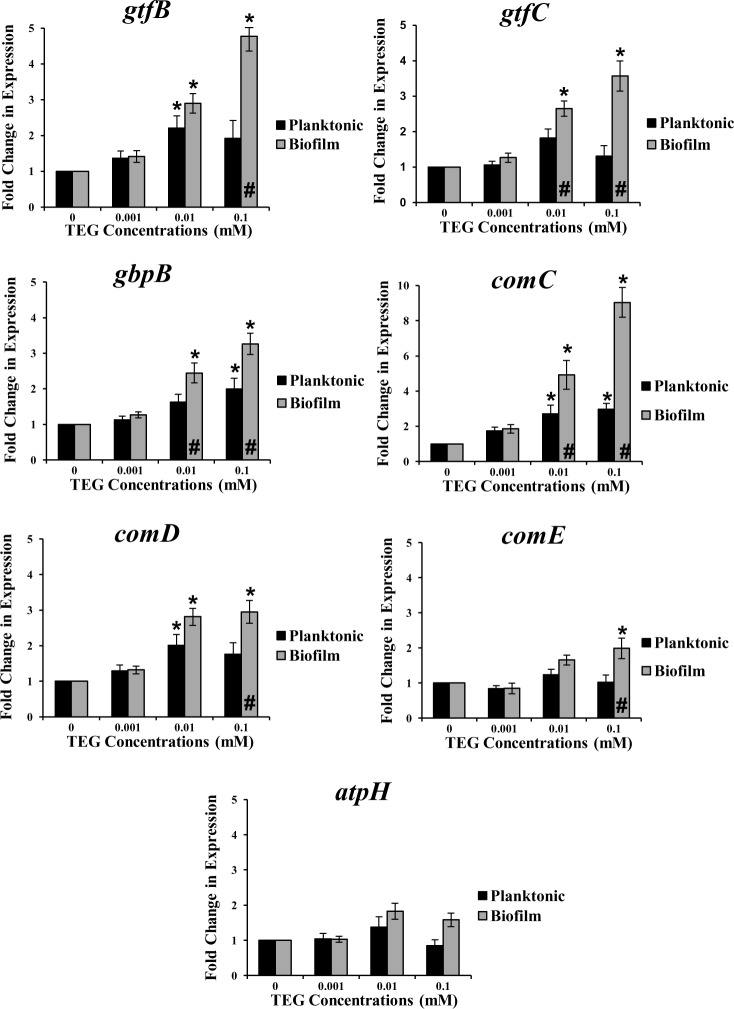
Relative expression of the *S*. *mutans* virulence genes: *gtfB*, *gtfC*, *gbpB*, *comC*, *comD*, *comE* and *atpH* for planktonic and biofilm growth conditions with different concentrations of TEG (0.001, 0.01 and 0.1 mM) at pH 5.5 relative to the no TEG control. * represents significant difference between individual TEG concentrations compared to the control (no TEG) in either growth mode (P<0.05). # Represents significant difference between biofilm and planktonic cultures at the same TEG concentration (P<0.001). Data are plotted with standard error of the mean (±SE), n = 4.

**Fig 2:**
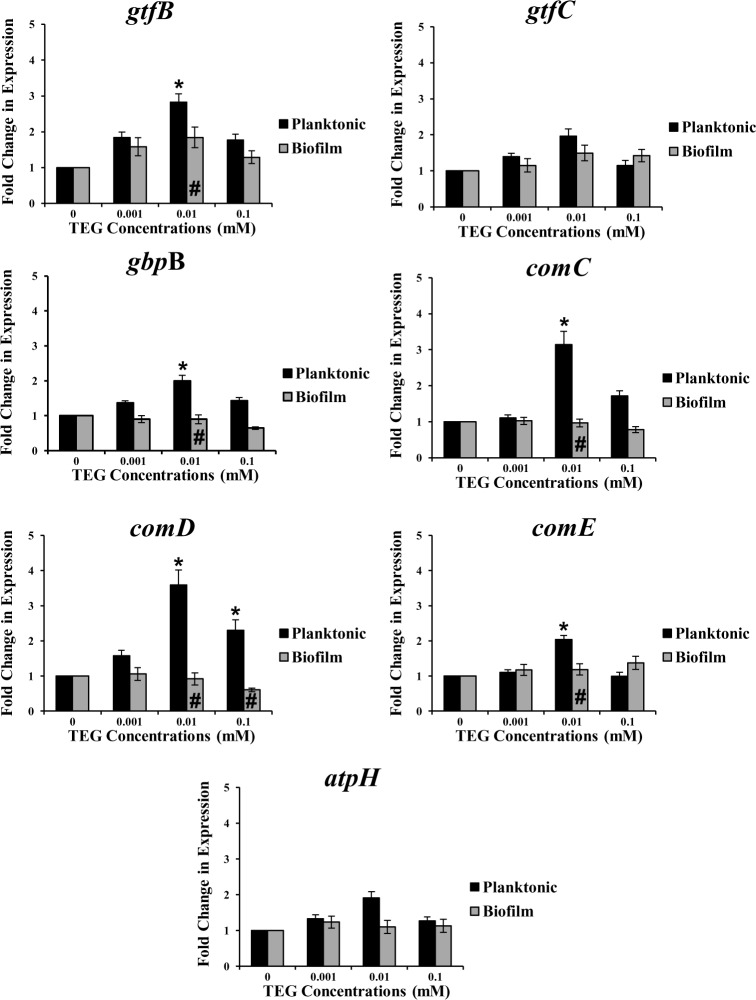
Relative expression of the *S*. *mutans* virulence genes: *gtfB*, *gtfC*, *gbpB*, *comC*, *comD*, *comE* and *atpH* for planktonic and biofilm growth conditions with different concentrations of TEG (0.001, 0.01 and 0.1 mM) at pH 7.0 relative to the no TEG control. * represents significant difference between individual TEG concentrations compared to the control (no TEG) in either growth mode (P<0.05). # represents significant difference between biofilm and planktonic cultures at the same TEG concentration (P<0.001). Data are plotted with standard error of the mean (±SE), n = 4.

Exposure of *S*. *mutans* UA159 to different concentrations of TEG at pH 5.5 significantly increased the expression of the four virulence genes, *gtfB*, *gbpB*, *comC* and *comD* at the concentration of 0.01 and/or 0.1 mM of TEG in both planktonic and biofilm cultures when compared to the no TEG control (P<0.05) (Fig 1). The fold change expression for the biofilm cultures was higher than the planktonic cells and was different for each gene, ranging from 2- to 9-fold (P<0.001). *GtfC* and *comE* were only up-regulated in biofilms (p<0.05) at 0.01 and 0.1 mM for *gtf*C and 0.1 mM for *comE*, respectively. *AtpH*, was not affected by TEG at any concentration in either growth mode (Fig 1). In contrast, at pH 7.0, expression of the selected genes in biofilms was not affected by TEG at any concentration, but, *gtfB*, *gbpB*, *com*C and *comE* were significantly (P<0.05) up-regulated in planktonic cultures at 0.01 mM TEG, and *comD* was up-regulated at both 0.01 and 0.1 mM (p<0.05) (Fig 2). The fold change levels were substantially lower at pH 7.0 when compared to the pH 5.5. Similarly to pH 5.5, *atpH* was not affected by TEG in either growth modes (Fig 2). Based on these findings, all genes were up-regulated more significantly in biofilms at cariogenic pH (5.5) in the presence of TEG. Hence, the remaining experiments, including knock-out and complemented experiments, proteomics and GTF enzyme activity, were conducted only with biofilms under acidic pH (5.5). Since 0.001 mM TEG had no effect on *S*. *mutans* virulence gene expression, this concentration was dropped from the remaining studies and a new higher concentration, 1.0 mM, was added to several of the subsequent studies [29].

### Effects of TEG on *S*. *mutans* UA159 *vicK* Knock-Out and Complemented Strains’ Gene Expression

**Fig 3:**
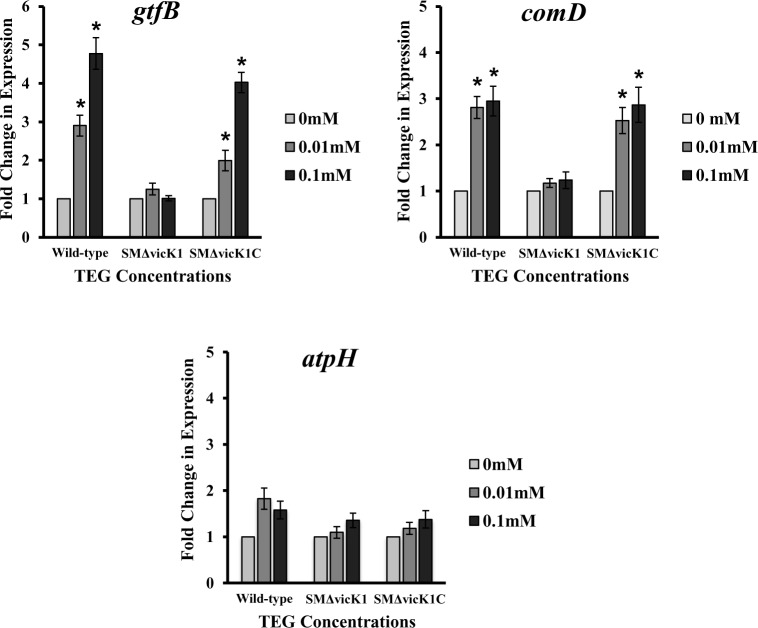
Relative expression of *gtfB*, *comD and atpH* in wild-type, knock-out (SMΔvicK1) and complemented (SMΔvicK1C) *vicK* strains of *S*. *mutans* UA159 in the presence of different concentrations of TEG (0.01 and 0.1 mM) at pH 5.5. One-way analysis of variance (ANOVA) and Tukey *post hoc* analyses were performed to determine the differences in gene expression between individual TEG concentration and the no-TEG control (P<0.05). Expression of the related genes in complemented strain was similar to that of wild-type. Data are plotted with standard error of the mean (±SE), n = 4.

Exposure of *S*. *mut*ans UA159 *vicK* knock-out strain (SMΔvicK1) to different concentrations of TEG resulted in no significant change (P>0.05) in the expression level for any of the selected virulence genes relative to the no TEG control. Data are presented for three representative genes, *gtfB* (implicated in biofilm formation), *comD* (associated with quorum sensing) and *atpH* (important to acid tolerance) in Fig 3. Exposure of *S*. *mutans* UA159 *vicK* complemented strain (SMΔvicK1C) to different concentrations of TEG resulted in a similar expression pattern of the aforementioned three representative genes to that of the wild-type strain (Fig 3). Both *gtfB* and *comD* were up-regulated by TEG at the same concentrations as the parent strain of 0.01 & 0.1 mM, while *atpH* was not affected by TEG at any concentration similar to what was observed for the wild-type strain (Fig 3).

### *S*. *mutans* Global Proteome Analysis in Response to TEG

**Fig 4:**
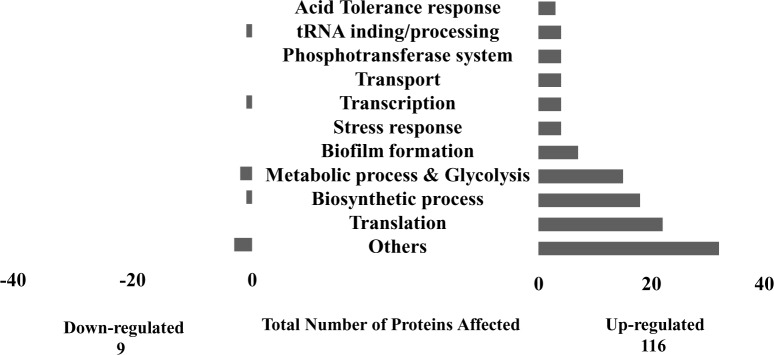
Distribution of *S*.*mutans* UA159 proteins into specific gene ontology (GO) of biological processes aftter exposure to TEG. A total number of 125 proteins were differentially expressed in *S*. *mutans* biofilm grown in the presence of different TEG concentrations (0.01, 0.1 and 1.0 mM), among which 116 proteins were more abundant and 9 proteins were less abundant compared to the no-TEG control.

The base-peak chromatogram for reversed-phase chromatography monitored by the mass spectrometer for all concentrations of TEG showed a consistent elution of protein/peptides in the range of 20 to 50 min (S1 Fig). A total number of 314 proteins have been identified (S1 Table), of which 125 proteins were differentially expressed in *S*. *mutans* biofilm grown in the presence of TEG, among which 116 proteins had enhanced expression (≥1.5 fold) and 9 proteins had diminished expression (≤ 0.5) fold compared to the control. The first step in the quantitative proteomic analysis by SIEVE was to promote an alignment of all mass spectrometry chromatograms (S1 Fig). Then the correlation coefficient score values were acquired for each mass spectrometric chromatogram and mean score values were calculated for each group. In the current study, one of the chromatograms from the no-TEG group was selected as the benchmark. All other chromatograms were compared with this chromatogram. The correlation coefficient mean values were 0.901 for 0.01 mM TEG group, 0.895 for 0.1 mM TEG group and, 0.841 for 1.0 mM TEG group. Interestingly, it was observed that by increasing the TEG concentration in each sample group, the alignment value (correlation coefficient value) became more distant from the default chromatogram group (S1 Fig). This observation suggested a change in quantity and quality of protein/peptides in a dose-dependent manner relative to the TEG concentrations, which means both number and amount of the proteins as wells as their time of elution were changing by increasing TEG concentrations. The distribution of proteins identified by gene ontology (GO) predicted and categorized a specific biological function for each protein and showed that the majority of proteins were associated with important functions such as energy metabolism, amino acid biosynthesis, transcription, translation, transport and binding (Fig 4). The organization was performed via proteomics website http://www.uniprot.org [52].

Exposure of *S*. *mutans* UA159 to different TEG concentrations resulted in a significantly higher (*P*<0.05) abundance of proteins involved in biofilm formation, specifically GtfB, GtfC, GbpB, ComD, ComE and SpaP (Table 3). As well, there was a TEG effect on proteins involved in acid-tolerance, which are different subunits of the F1F0-ATPase proton translocating pump (ATPA, subunit α, ATPD, subunit β and AtpH, subunit *C*) (Table 4). The findings also identified TEG effects on proteins involved in stress response (DnaK, RecA, GroL and ClpX) (Table 5), and proteins encoding the Phosphoenolpyruvate: phosphotransferase sugar transport system (PEP:PTS), including: *ptsI* encodes EI; *mtlF* encodes EII; *SMU1879* and *SMU1960c* encodes EIID ^man^ and EIIB ^man^ respectively (Table 6). Further, another sixty-six individual proteins differing in abundance by two-fold or more including sixteen proteins involved in metabolic processes and glycolysis, five proteins involved in transcription, five proteins involved in RNA binding and processing, twenty-two proteins involved in translation, nineteen proteins involved in amino acid biosynthesis, four proteins involved in transport, and finally thirty-four proteins with their functions either uncharacterized or that did not belong to any identified categories (Tables A-G in S1 File).

**Table 3:** Biofilm-related proteins from *S*. *mutans* UA159 grown in the presence of TEG at pH 5.5.

Gene name	Protein name	Protein function	Ratio 1mM/0mM	P	Ratio 0.1mM/0mM	P	Ratio 0.01mM/0mM	P
*gtfB*	GtfB	Glucan biosynthetic process	2.34*	0.01	1.57*	0.01	2.54*	0.01
*gtfC*	GtfC	Glucan biosynthetic process	1.55*	0.01	1.41	0.12	0.79	0.07
*gbpB*	GbpB	single-species biofilm formation on inanimate substrate	1.54*	0.01	1.09	0.09	0.65	0.85
*comC*	ComC	Multiorganism process, biological adhesion	1.12	0.02	1.10	0.03	0.99	0.02
*comD*	ComD	Single-organism process, cellular process, metabolic process	0.90	0.06	1.90*	0.04	0.99	0.04
*comE*	ComE	signaling,biological regulation, single-organism process	1.76*	0.01	1.87	0.06	2.40*	0.05
*spaP*	Cell-surface antigen I/II	(Surface protein antigen implicated in dental caries)	1.92*	0.05	0.61	0.02	0.69	0.01

*Represents P<0.05

**Table 4:** Acid tolerance-response proteins from *S*. *mutans* UA159 grown in the presence of TEG at pH 5.5.

Gene name	Protein name	Protein function	Ratio 1mM/0mM	P	Ratio 0.1mM/0mM	P	Ratio 0.01mM/0mM	P
*atpA*	ATP synthase F1, alpha subunit	ATP hydrolysis coupled proton transport,plasma membrane ATP synthesis coupled proton transport	0.91	0.05	1.94*	0.01	0.62	0.01
*atpD*	ATP synthase F1, beta subunit	ATP hydrolysis coupled proton transport,plasma membrane ATP synthesis coupled proton transport	0.96	0.01	1.58*	0.05	0.53	0.02
*atpH*	F1F0 membrane-bound proton-translocating ATPase c subunit	hydrogen ion transmembrane transporter activity	2.13*	0.02	1.57	0.13	2.56	0.21

*Represents P<0.05

**Table 5:** Stress-response proteins from *S*. *mutans* UA159 grown in the presence of TEG at pH 5.5.

Gene name	Protein name	Protein function	Ratio 1mM/0mM	P	Ratio 0.1mM/0mM	P	Ratio 0.01mM/0mM	P
*DnaK*	Chaperone protein DnaK	protein folding,response to stress	1.30	0.92	1.65*	0.005	2.20*	0.05
*recA*	Protein RecA	DNA recombination, DNA repair, SOS response	1.98*	0.02	1.71*	0.028	1.67*	0.01
*groL*	60 kDa chaperonin	protein refolding	1.67*	0.01	1.82*	0.000	2.36*	0.04
*clpX*	ATP-dependent Clp protease, ATP-binding subunit	protein folding	2.15*	0.01	1.61	0.088	2.07*	0.01

*Represents P<0.05

**Table 6:** Carbohydrate transport proteins from *S*. *mutans* UA159 grown in the presence of TEG at pH5.5.

Gene name	Protein name	Protein function	Ratio 1mM/0mM	P	Ratio 0.1mM/0mM	P	Ratio 0.01mM/0mM	P
*ptsI*	Phosphoenolpyruvate-protein phosphotransferase	PEP:PTS Core phosphotransfer protein	2.09*	0.01	2.10*	0.05	1.43	0.01
*mtlF*	Phosphotransferase system enzyme II	PEP:PTS permease	1.79*	0.02	1.84*	0.02	2.21*	0.01
*SMU_1879*	Mannose-specific phosphotransferase system component IID	PEP:PTS permease/regulatory protein	1.99*	0.01	1.99*	0.01	0.86	0.04
*SMU_1960c*	PTS system protein, mannose-specific IIB component	PEP:PTS regulatory protein	1.84*	0.01	1.85*	0.01	2.36*	0.01

*Represents P<0.05

### Effects of TEG on GTF Enzyme Activity

**Fig 5:**
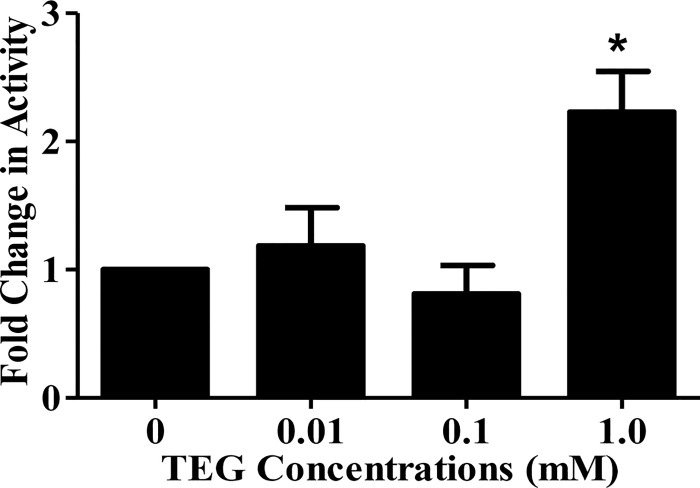
Effects of different concentrations of TEG (0.01, 0.1 and 1.0 mM) on *S*. *mutans* UA159 glucosyltransferase activity. The total amount of insoluble glucan synthesized by biofilm cells grown in the presence of 1 mM TEG was significantly increased compared to the no-TEG control (P<0.05). Data are plotted with standard error of the mean (±SE), n = 4.

The total amount of insoluble glucan synthesized by *S*. *mutans* UA159 cell-associated GTF grown in the presence or absence of different concentrations of TEG was significantly higher P<0.05) for cells grown in the presence of 1.0 mM TEG *vs*. no TEG (Fig 5). There was no effect related to the 0.01 and 0.1 mM concentrations of TEG on GTF enzyme activity (Fig 5).

## Discussion

The findings of the present study suggest that the degradation product from the most common diluent resin composite monomer, TEGDMA, in concentrations relevant to *in vivo* conditions [29], may contribute to *S*. *mutans* cariogenic potential by up-regulating several virulence genes/related proteins and activating specific adaptation mechanisms such as biofilm formation, acid tolerance, optimization/modulation of the PEP:PTS system, and stress response pathways. At the cariogenic pH of 5.5, up-regulation of *gtfB*, *gtfC*, *gbpB*, *comC*, *comD* and *comE* was more significant for biofilms when compared to the planktonic cells. These are relevant findings since both biofilm and pH 5.5 are conditions for development of dental caries *in vivo*. The data also provided further insight into the molecular pathways involved in *S*. *mutans* response to the hydrophilic TEGDMA derived degradation product, TEG. This was emphasized by evaluating the role of a two-component signal transduction system sensor protein encoded by *vicK*, which has been shown to have a regulatory control on the *comCDE* system and also influences *gtfB*, *gtfC*, *gbpB* expression and acid tolerance in *S*. *mutans* [40–43].

TEG is a small molecule with a low molecular weight of 150.17 g/mol and is the ether glycol portion of the TEGDMA monomer. It is characterised by two hydroxyl groups along with two ether linkages, which contribute to its high water solubility, hygroscopicity, solvent properties and reactivity with many organic compounds [53]. Due to its lipophilic nature, TEGDMA, the precursor of TEG, can easily penetrate the cytosol and lipid compartment of mammalian cells [54, 55]. There have been a few studies regarding the metabolism of polyethylene glycol monomers in bacteria, indicating the oxidation of these monomers to an aldehyde and carboxylic acid by aldehyde or alcohol dehydrogenase, followed by cleavage of ether bond resulting in a polyglycol molecules that are reduced by one glycol unit [54, 56, 57].

TEG has been used for a variety of applications in industry including natural gas dehydration and as a humectant, solvent, and chemical intermediate in the synthesis of resins, plasticizers, lubricants and polyurethanes [53]. In the 1940s TEG vapor or mist was first introduced for disinfection purposes, especially in hospital wards [58]. A few studies have shown TEG to be bactericidal for Beta hemolytic *Streptococcus*, *Streptococcus pneumoniae*, *Streptococcus pyogenes*, Influenza A virus and *Penicillium notatum* fungi [59–64]. It was also found that concentrated polyethylene glycol 400 (PEG 400) solutions have antibacterial activity against several pathogenic bacteria including *Klebsiella pneumoniae*, *Pseudomonas aeruginosa*, *Escherichia coli*, and *Staphylococcus aureus* [65].

Although these studies indicated that TEG had some bactericidal activity towards several bacterial species, most of the works have only demonstrated the antimicrobial activity of TEG in its vapor form against airborne, solution suspended, and surface bound microbes rather than cariogenic bacterial biofilms such as *S*. *mutans*. In addition, higher dosages of bactericide are needed to eradicate bacteria existing in biofilms compared to their planktonic counterparts due to the protective nature of biofilms [66–69]. The *in vivo*-relevant concentrations of TEG that have been used in the current study (0.001–1 mM) are far from the concentrations that were used in cases where TEG demonstrated antibacterial activity against non-cariogenic bacteria (e.g. Robertson et al. 250 mM or Chirife et al. 4000 mM) [62, 65]. Further, Khalichi *et al*. [70] reported that TEG at the concentration range of 0.5–10.0 mM significantly stimulated the growth of two *S*. *mutans* strains (NG8 and JH1005) only at pH 5.5. These findings suggest that the effect of TEG on bacteria is dosage- and pH- dependant and varies among different bacterial species.

It has been shown that anaerobic bacteria are capable of degrading glycol monomers using oligo- and polyethylene glycols and ether alcohols as their carbon source especially in minimum media [56, 71]. In addition, the small size and hydrophilic nature of TEG can facilitate its mobility within the biofilms’ exopolysaccharide matrix, activating TCSTs and the resultant gene expression response. The knock-out and complemented strain experiments in the present study, strongly suggest that exposure of *S*. *mutans* biofilms to TEG results in up-regulation of virulence-associated genes via *vicRK* signalling pathway.

The more pronounced up-regulation of the virulence-associated genes at acidic pH *vs*. neutral pH by TEG could be related to the fact that low pH (5.5) triggers changes in the bacterial cell membrane fatty acid composition that can affect membrane permeability [72] and would results in even easier penetration of TEG from the environment. Further, TEG exhibits the properties of a weak acid (pKa of 15.12), therefore, its undissociated form at acidic pH might facilitate its penetration into the bacterial membrane compared to neutral pH [73, 74], where it can further react and degrade, affecting various signaling pathways such as stress response, carbohydrates transport and acid tolerance. The greater effect of TEG on biofilms *vs*. planktonic growth mode can be explained by the close proximity of the cells in the former growth mode, shortening and enhancing the response time to BBP, as well as making the effective concentration of the BBP higher due to a diffusion-barrier effect of the biofilm [12].

Among the *S*. *mutans* virulence attributes, the ability of this bacterium to produce extracellular polysaccharide from dietary carbohydrates has been demonstrated to significantly enhance its cariogenicity [30]. *GtfB* and *gtfC* encode the enzymes glucosyltransferase-*B* (GTF-I) and-*C* (GTF-SI) respectively, which are responsible for the synthesis of water-insoluble glucans that function in the adhesion and accumulation of the bacteria on the tooth surfaces. Mutant strains of *S*. *mutans* defective in *gtf* genes, especially *gtfB* and *gtfC*, are far less cariogenic than the parent strains *in vivo* [38]. Oral bacterial aggregation is also mediated by interactions between surface-associated glucan-binding proteins (GBPs) that adhere to glucans, thereby promoting plaque formation [39, 75]. GbpB is also associated with *S*. *mutans* virulence because immunization with this protein mediates protection against dental caries in animal models [76]. In addition, GbpB appears to play an essential function in *S*. *mutans* viability and has also been described as a stress-response protein [77]. Hence, the upregulation of these genes by TEG suggests that there may be an increase in the virulence of the bacteria.

The proteomics findings demonstrated the enhanced expression of biofilm-related proteins in the presence of TEG in *S*. *mutans* biofilms at cariogenic pH (5.5). Up-regulation of *gtfB*, *gtfC* and *gbpB* and their corresponding proteins by TEG could have a significant impact on *S*. *mutans* cariogenicity by making thicker and stickier biofilm and potentially contributes in resin composite restoration failure [78–80].

The gene expression analysis demonstrated the greatest change in fold expression for *comC* with a nine-fold increase in mRNA levels in the presence of 0.1 mM TEG when compared to the no-TEG control in biofilm grown cells at pH 5.5. In addition, *comD* and *comE* were up-regulated by TEG at 0.01 and/or 0.1 mM in biofilms at pH 5.5 relative to the control. In *S*. *mutans comDE* TCSTS together with *comC*, which encodes competence stimulating peptide (CSP), regulate cellular processes and many virulence factors including genetic transformation, biofilm formation, acidogenicity, aciduricity, cell viability and bacteriocin production in response to environmental cues [81]. Importantly, the proteomics findings regarding the biofilm-associated proteins, GtfB, GtfC, GbpB, ComC, ComD, ComE are in agreement with the gene expression results for the respective genes. The fold change in protein synthesis and gene expression was not at the same magnitude in all cases, however the trends were conserved. The discordance in specific dose responses could be due to differences in the sensitivity of the methods, post-translational modifications of proteins and the status of mRNA stability and degradation [80, 82, 83]. Of particular note, the presence of 1.0 mM TEG also increased the protein abundance of SpaP, which is the major *S*. *mutans* surface receptor (known as antigen I/II and wall-associated antigen), that contributes to sucrose-independent adhesion [28, 84–86]. SpaP was one of the first gene products linked to adherence of *S*. *mutans* to saliva coated surfaces [86].

A very significant finding in this work was the GTF enzyme activity results, which indicated that the total rate of insoluble glucan synthesis by *S*. *mutans* UA159 biofilms grown in the presence of 1.0 mM TEG was significantly higher (P<0.05) compared to the no-TEG control biofilms. These findings suggest that TEG can affect *S*. *mutans* virulence factors at three different molecular levels (mRNA, protein and enzyme activity), which can eventually result in production of thicker exopolysaccharide glucan as a major contributor in *S*. *mutans* virulence [78–80].

The findings of this study provide a mechanistic explanation to the role of biomaterials-bacterial interaction in the pathogenesis of secondary caries, by uncovering underlying signalling pathways, specifically the *vicRK* regulatory system and its dependence on TEG with respect to the *S*. *mutans* virulence genes expression. This TCSTS plays important roles in the regulation of genetic competence, biofilm formation, and acid tolerance by regulating *comCDE* system and biofilm associated genes, *gtfB*, *gtfC*, *gbpB*, in *S*. *mutans* [39–43].

Oral biofilms are regularly subjected to acid stress as a result of the accumulation of acidic end-products from bacterial carbohydrate metabolism. *S*. *mutans*, the major cariogenic bacteria in plaque, is adapted to function well at low pH values and its cariogenicity is closely related to its acid tolerance [87]. The membrane-bound F1F0-ATPase pump is the primary mechanism of proton extrusion in *S*. *mutans*. To maintain pH homeostasis, the F1F0-ATPase export H^+^ as the growth conditions become more acidic [88]. The proteomic data indicated that three subunits of F1F0-ATPase pump [AtpA (subunit α), AtpD (subunit β) and AtpH (subunit *C*)] had a significantly increased abundance of related proteins upon exposure to different concentrations of TEG. While AtpA and AtpD were induced by 0.1 mM TEG, AtpH was only induced at 1.0 mM TEG, which is in agreement with the gene expression analysis indicating no significant effect at lower concentrations of TEG (0.01 and 0.1 mM) for AtpH. On the other hand, both AtpA and AtpD were downregulated at 0.01 mM. The observed variances for the different protein products could reflect the heterogeneity of biofilm cells, which might have resulted in different response with different concentrations of TEG in some cases [50]. Such findings suggest that *S*. *mutans* may have the ability to enhance acid adaptation and survival after exposure to dental composite degradation product.

To cope with the environmental stresses, *S*. *mutans* has developed several strategies that allow it to grow under harsh conditions. For example *S*. *mutans* induces the expression of a group of proteins that are either chaperones or proteases [89]. Unfolded or misfolded proteins accumulate inside the cell due to stress, and molecular chaperones are required to proper folding and assembly of those aberrant proteins [89]. Proteases are involved in the degradation of the proteins, not only under stress conditions, but also under normal growth conditions [89]. Stress responsive proteins, DnaK, RecA, GroL and ClpX were among the proteins that were more abundant (by two-fold or greater) in at least one of the three TEG concentrations when compared to the no TEG control. The production of stress proteins including DnaK, RecA, GroL and ClpX is central to the tolerance of environmental insults by microorganisms [90]. DnaK is known to be induced in response to multiple stresses such as acid shock and carbon starvation [91]. Lowering the DnaK level results in impaired capacity of *S*. *mutans* to form biofilm in the presence of glucose [92]. In other work it has been shown that the recombinase A (RecA) protein is involved in the homologous recombination of GtfB and GtfC in *S*. *mutans* [93]. RecA is also contributor to late steps of competence development as well as biofilm formation and acid stress survival [87, 94]. Both DnaK and GroL can regulate signal transduction pathways by controlling the stability and activities of transcriptional regulators and protein kinases [95, 96].

CLP proteins are responsible for degrading proteins that cannot be folded by molecular chaperones [97, 98]. ClpX has been shown to modulate the expression of important virulence attributes of *S*. *mutans* including biofilm formation, viability and acid survival [99]. Expression of genes involved in biofilm formation, competence and mutacin production was down-regulated in ClpX deficient mutant strain [99]. The data in the current study suggests that ClpX presents a high level of abundance (more than two fold) when 0.01 and 1.0 mM of TEG were added to the bacterial cultures. Collectively, these findings suggest that TEG can enhance *S*. *mutans* ability to adapt to environmental stresses by modulating stress-response pathways in order to survive and persist in cariogenic biofilms.

The proteomics results also demonstrated that TEG has the ability to induce the expression of the PEP:PTS, which is the main carbohydrate transport system in oral streptococci [90]. Carbohydrate transport and metabolism can be used as fuel for the downstream energy generating pathways such as glycolysis [100]. Given the important role of the PEP:PTS system in *S*. *mutans* sugar transport, metabolism and global regulatory effect on gene expression, specifically controlling biofilm formation and acid tolerance [101, 102], up-regulation of different components of this system by TEG is a very significant finding.

In general, the findings indicate global changes in protein synthesis by *S*. *mutans* biofilms in response to the dental composite degradation product TEG, which may contribute to *S*. *mutans* successful fitness and establishment in diverse cariogenic biofilm. These findings may provide an explanation for the higher failure rate due to increased presence of secondary caries, and increased frequency of replacement of resin composite restorations compared to amalgam [103]. More importantly, the findings signal a call to the clinical community to insist that commercial manufacturers for resin composites exercise more due diligence on the biological characterization of their products and related degradation by-products, and to refrain from referring to current materials as biologically inert dental materials.

This *in vitro* study used only one of the multiple bacterial species found in dental plaque and growth conditions for biofilms were simplified, and as such, the data cannot reproduce all of the complex *in vivo* conditions of plaque. However, the findings of the current study provide much new insight into the potential mechanisms behind recurrent caries and contribution of resin composite degradation products to the failure of resin composite restorations. In addition, this simplified *in vitro* system can provide the opportunity to examine direct effect(s) of specific molecules such as TEG and other resin composite components and/or degradation products on bacterial physiological responses, independent of other complex interactions *in vivo*. Future studies could investigate the influence of the resin composite degradation products on the pathogenic potential of cariogenic oral bacteria in a multi-species biofilm under both *in vitro* and *in vivo* conditions.

## Supporting Information

S1 Fig Base-peak chromatogram for four different TEG concentrations showing a consistent elution of protein/peptides range from 20 to 50 min, which suggests a change in quantity and quality of protein/peptides by increasing TEG concentrations. Blue (1.0 mM), red (0.1 mM), violet (0.01mM) and green (0.00 mM).
(TIF)

S1 File **Tables A-G:** Other differentially expressed proteins following exposure to TEG.
(PPTX)

S1 Table Effects of TEG on *S*. *mutans* biofilm global protein expression at cariogenic pH (5.5). (XLSX)
