# Supplementary figures and images for: Triethylene Glycol Up-Regulates Virulence-Associated Genes and Proteins in Streptococcus mutans
Source: PLoS One. 2016 Nov 7;11(11):e0165760. doi: 10.1371/journal.pone.0165760 (PMC5098727; doi:10.1371/journal.pone.0165760)

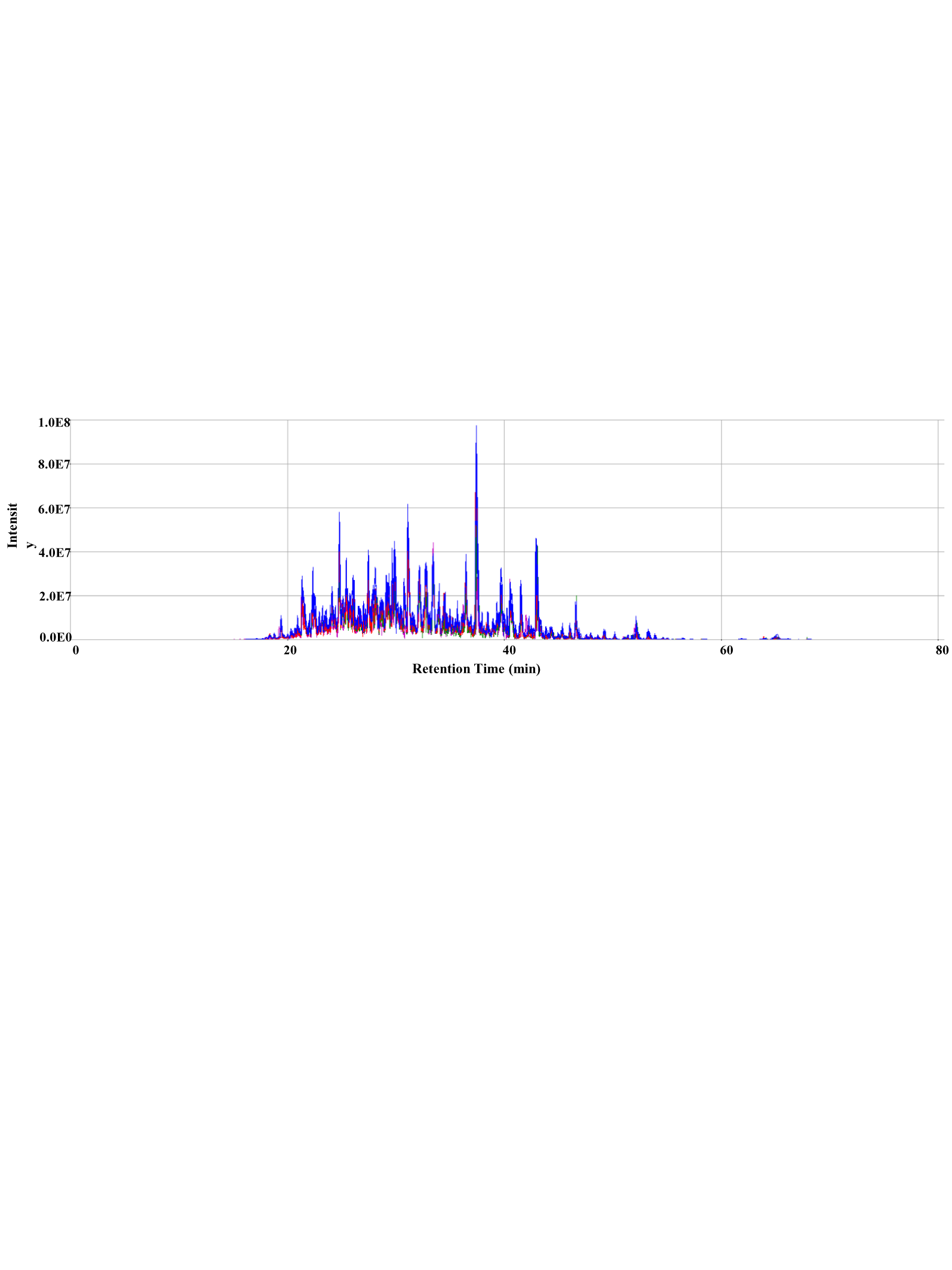

Supplement: S1 Fig — Blue (1.0 mM), red (0.1 mM), violet (0.01mM) and green (0.00 mM). (TIF) [file pone.0165760.s001.tif]
