# Supplementary material for: Triethylene Glycol Up-Regulates Virulence-Associated Genes and Proteins in Streptococcus mutans
Source: PLoS One. 2016 Nov 7;11(11):e0165760. doi: 10.1371/journal.pone.0165760 (PMC5098727; doi:10.1371/journal.pone.0165760)
Supplement: S1 File — Tables A-G: Other differentially expressed proteins following exposure to TEG. (PPTX) [file pone.0165760.s002.pptx]

## Slide 1
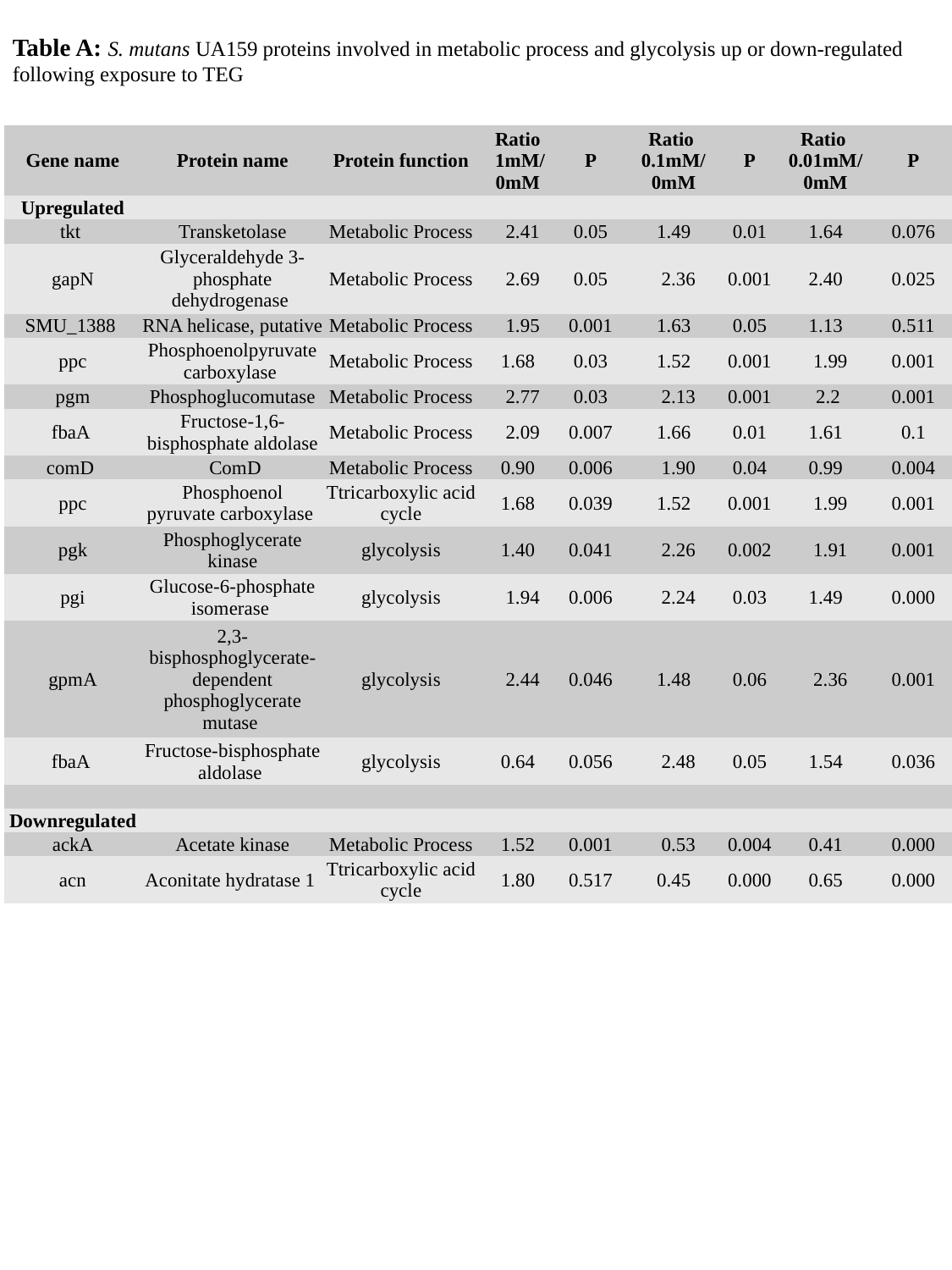

Table A: S. mutans UA159 proteins involved in metabolic process and glycolysis up or down-regulated following exposure to TEG
| Gene name | Protein name | Protein function | Ratio 1mM/ 0mM | P | Ratio 0.1mM/ 0mM | P | Ratio 0.01mM/ 0mM | P |
| --- | --- | --- | --- | --- | --- | --- | --- | --- |
| Upregulated | | | | | | | | |
| tkt | Transketolase | Metabolic Process | 2.41 | 0.05 | 1.49 | 0.01 | 1.64 | 0.076 |
| gapN | Glyceraldehyde 3-phosphate dehydrogenase | Metabolic Process | 2.69 | 0.05 | 2.36 | 0.001 | 2.40 | 0.025 |
| SMU\_1388 | RNA helicase, putative | Metabolic Process | 1.95 | 0.001 | 1.63 | 0.05 | 1.13 | 0.511 |
| ppc | Phosphoenolpyruvate carboxylase | Metabolic Process | 1.68 | 0.03 | 1.52 | 0.001 | 1.99 | 0.001 |
| pgm | Phosphoglucomutase | Metabolic Process | 2.77 | 0.03 | 2.13 | 0.001 | 2.2 | 0.001 |
| fbaA | Fructose-1,6-bisphosphate aldolase | Metabolic Process | 2.09 | 0.007 | 1.66 | 0.01 | 1.61 | 0.1 |
| comD | ComD | Metabolic Process | 0.90 | 0.006 | 1.90 | 0.04 | 0.99 | 0.004 |
| ppc | Phosphoenol pyruvate carboxylase | Ttricarboxylic acid cycle | 1.68 | 0.039 | 1.52 | 0.001 | 1.99 | 0.001 |
| pgk | Phosphoglycerate kinase | glycolysis | 1.40 | 0.041 | 2.26 | 0.002 | 1.91 | 0.001 |
| pgi | Glucose-6-phosphate isomerase | glycolysis | 1.94 | 0.006 | 2.24 | 0.03 | 1.49 | 0.000 |
| gpmA | 2,3-bisphosphoglycerate-dependent phosphoglycerate mutase | glycolysis | 2.44 | 0.046 | 1.48 | 0.06 | 2.36 | 0.001 |
| fbaA | Fructose-bisphosphate aldolase | glycolysis | 0.64 | 0.056 | 2.48 | 0.05 | 1.54 | 0.036 |
| | | | | | | | | |
| Downregulated | | | | | | | | |
| ackA | Acetate kinase | Metabolic Process | 1.52 | 0.001 | 0.53 | 0.004 | 0.41 | 0.000 |
| acn | Aconitate hydratase 1 | Ttricarboxylic acid cycle | 1.80 | 0.517 | 0.45 | 0.000 | 0.65 | 0.000 |

## Slide 2
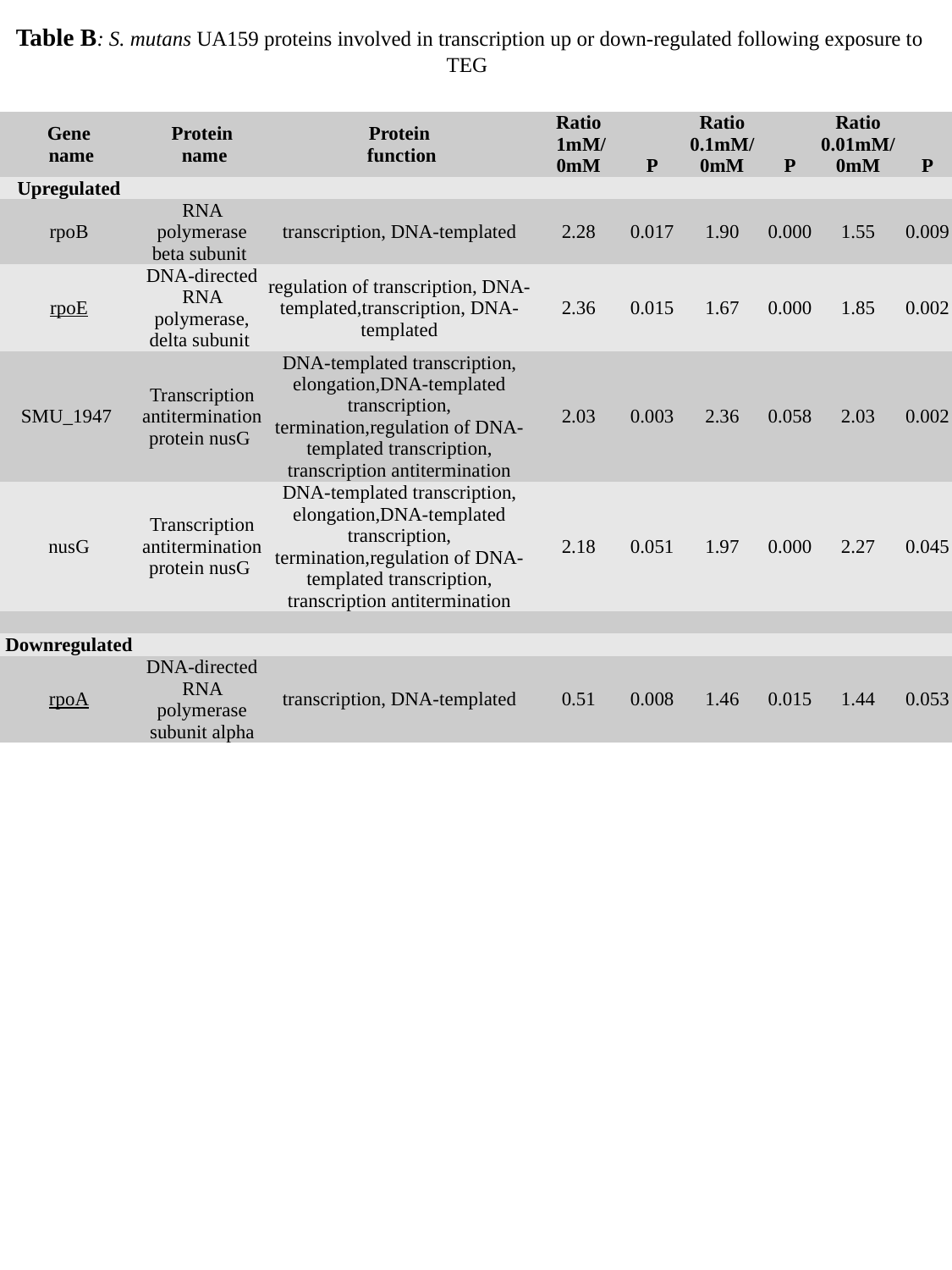

Table B: S. mutans UA159 proteins involved in transcription up or down-regulated following exposure to TEG
| Gene name | Protein name | Protein function | Ratio 1mM/ 0mM | P | Ratio 0.1mM/ 0mM | P | Ratio 0.01mM/ 0mM | P |
| --- | --- | --- | --- | --- | --- | --- | --- | --- |
| Upregulated | | | | | | | | |
| rpoB | RNA polymerase beta subunit | transcription, DNA-templated | 2.28 | 0.017 | 1.90 | 0.000 | 1.55 | 0.009 |
| rpoE | DNA-directed RNA polymerase, delta subunit | regulation of transcription, DNA-templated,transcription, DNA-templated | 2.36 | 0.015 | 1.67 | 0.000 | 1.85 | 0.002 |
| SMU\_1947 | Transcription antitermination protein nusG | DNA-templated transcription, elongation,DNA-templated transcription, termination,regulation of DNA-templated transcription, transcription antitermination | 2.03 | 0.003 | 2.36 | 0.058 | 2.03 | 0.002 |
| nusG | Transcription antitermination protein nusG | DNA-templated transcription, elongation,DNA-templated transcription, termination,regulation of DNA-templated transcription, transcription antitermination | 2.18 | 0.051 | 1.97 | 0.000 | 2.27 | 0.045 |
| | | | | | | | | |
| Downregulated | | | | | | | | |
| rpoA | DNA-directed RNA polymerase subunit alpha | transcription, DNA-templated | 0.51 | 0.008 | 1.46 | 0.015 | 1.44 | 0.053 |

## Slide 3
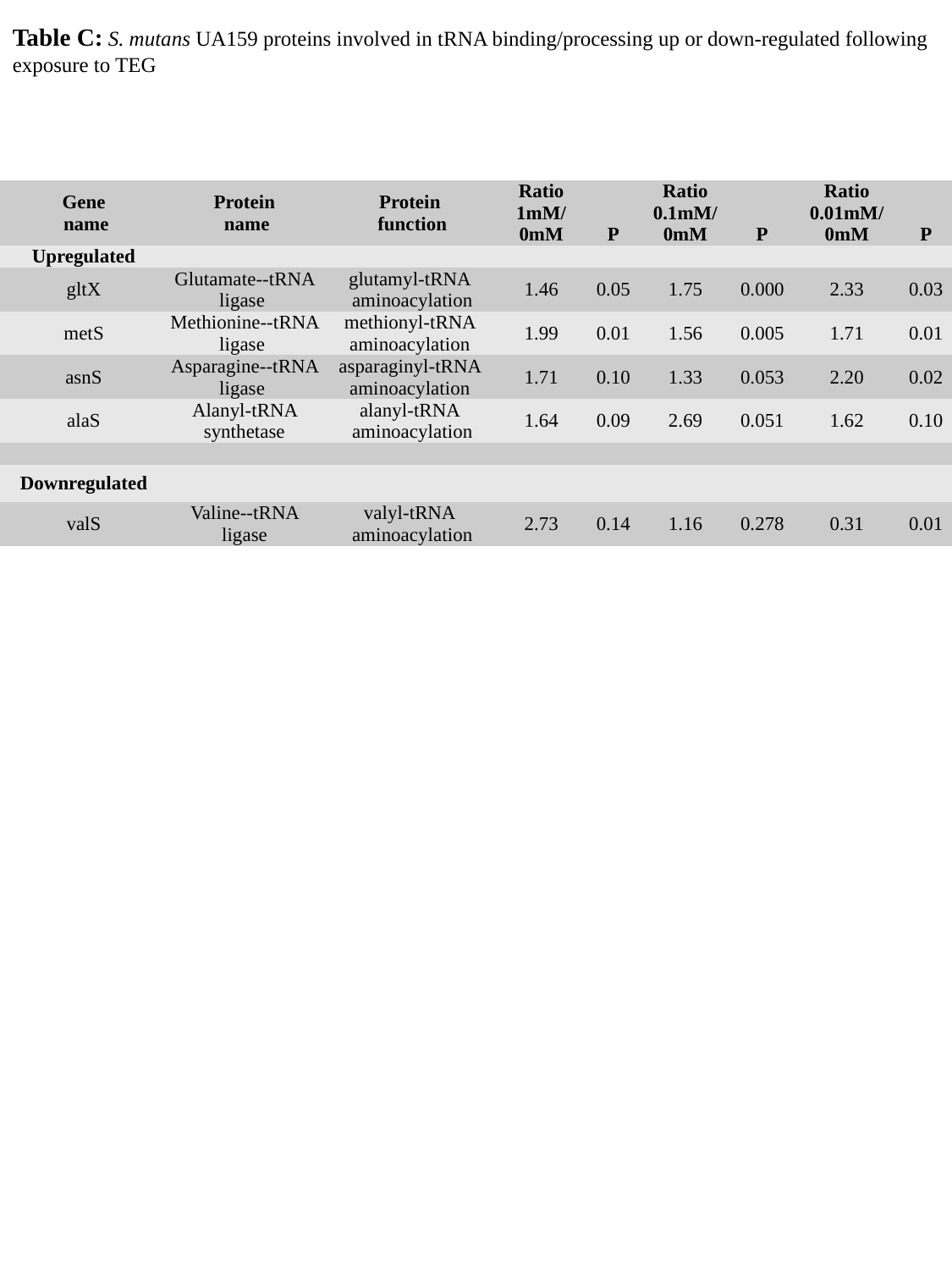

Table C: S. mutans UA159 proteins involved in tRNA binding/processing up or down-regulated following exposure to TEG
| Gene name | Protein name | Protein function | Ratio 1mM/ 0mM | P | Ratio 0.1mM/ 0mM | P | Ratio 0.01mM/ 0mM | P |
| --- | --- | --- | --- | --- | --- | --- | --- | --- |
| Upregulated | | | | | | | | |
| gltX | Glutamate--tRNA ligase | glutamyl-tRNA aminoacylation | 1.46 | 0.05 | 1.75 | 0.000 | 2.33 | 0.03 |
| metS | Methionine--tRNA ligase | methionyl-tRNA aminoacylation | 1.99 | 0.01 | 1.56 | 0.005 | 1.71 | 0.01 |
| asnS | Asparagine--tRNA ligase | asparaginyl-tRNA aminoacylation | 1.71 | 0.10 | 1.33 | 0.053 | 2.20 | 0.02 |
| alaS | Alanyl-tRNA synthetase | alanyl-tRNA aminoacylation | 1.64 | 0.09 | 2.69 | 0.051 | 1.62 | 0.10 |
| | | | | | | | | |
| Downregulated | | | | | | | | |
| valS | Valine--tRNA ligase | valyl-tRNA aminoacylation | 2.73 | 0.14 | 1.16 | 0.278 | 0.31 | 0.01 |

## Slide 4
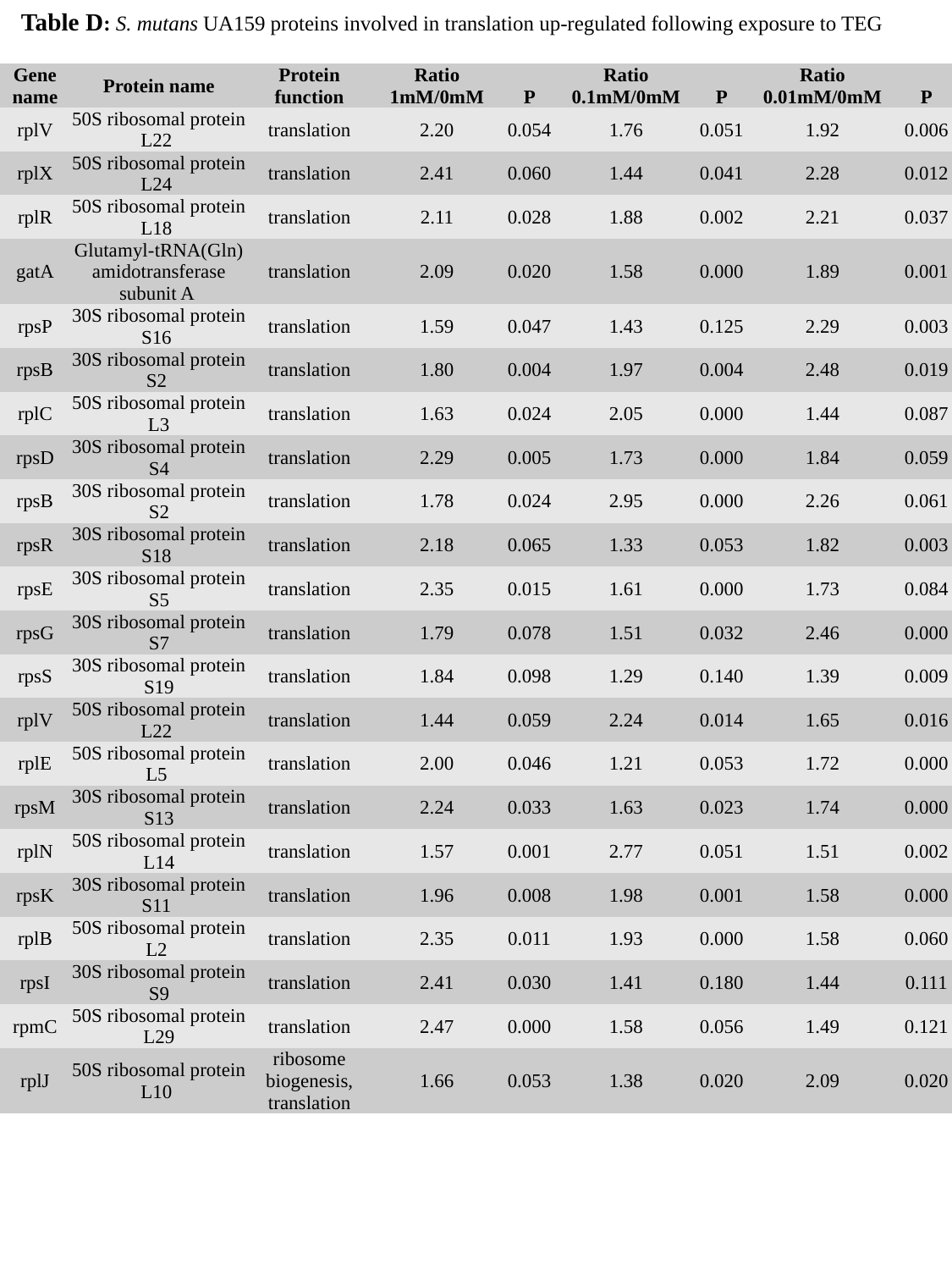

Table D: S. mutans UA159 proteins involved in translation up-regulated following exposure to TEG
| Gene name | Protein name | Protein function | Ratio 1mM/0mM | P | Ratio 0.1mM/0mM | P | Ratio 0.01mM/0mM | P |
| --- | --- | --- | --- | --- | --- | --- | --- | --- |
| rplV | 50S ribosomal protein L22 | translation | 2.20 | 0.054 | 1.76 | 0.051 | 1.92 | 0.006 |
| rplX | 50S ribosomal protein L24 | translation | 2.41 | 0.060 | 1.44 | 0.041 | 2.28 | 0.012 |
| rplR | 50S ribosomal protein L18 | translation | 2.11 | 0.028 | 1.88 | 0.002 | 2.21 | 0.037 |
| gatA | Glutamyl-tRNA(Gln) amidotransferase subunit A | translation | 2.09 | 0.020 | 1.58 | 0.000 | 1.89 | 0.001 |
| rpsP | 30S ribosomal protein S16 | translation | 1.59 | 0.047 | 1.43 | 0.125 | 2.29 | 0.003 |
| rpsB | 30S ribosomal protein S2 | translation | 1.80 | 0.004 | 1.97 | 0.004 | 2.48 | 0.019 |
| rplC | 50S ribosomal protein L3 | translation | 1.63 | 0.024 | 2.05 | 0.000 | 1.44 | 0.087 |
| rpsD | 30S ribosomal protein S4 | translation | 2.29 | 0.005 | 1.73 | 0.000 | 1.84 | 0.059 |
| rpsB | 30S ribosomal protein S2 | translation | 1.78 | 0.024 | 2.95 | 0.000 | 2.26 | 0.061 |
| rpsR | 30S ribosomal protein S18 | translation | 2.18 | 0.065 | 1.33 | 0.053 | 1.82 | 0.003 |
| rpsE | 30S ribosomal protein S5 | translation | 2.35 | 0.015 | 1.61 | 0.000 | 1.73 | 0.084 |
| rpsG | 30S ribosomal protein S7 | translation | 1.79 | 0.078 | 1.51 | 0.032 | 2.46 | 0.000 |
| rpsS | 30S ribosomal protein S19 | translation | 1.84 | 0.098 | 1.29 | 0.140 | 1.39 | 0.009 |
| rplV | 50S ribosomal protein L22 | translation | 1.44 | 0.059 | 2.24 | 0.014 | 1.65 | 0.016 |
| rplE | 50S ribosomal protein L5 | translation | 2.00 | 0.046 | 1.21 | 0.053 | 1.72 | 0.000 |
| rpsM | 30S ribosomal protein S13 | translation | 2.24 | 0.033 | 1.63 | 0.023 | 1.74 | 0.000 |
| rplN | 50S ribosomal protein L14 | translation | 1.57 | 0.001 | 2.77 | 0.051 | 1.51 | 0.002 |
| rpsK | 30S ribosomal protein S11 | translation | 1.96 | 0.008 | 1.98 | 0.001 | 1.58 | 0.000 |
| rplB | 50S ribosomal protein L2 | translation | 2.35 | 0.011 | 1.93 | 0.000 | 1.58 | 0.060 |
| rpsI | 30S ribosomal protein S9 | translation | 2.41 | 0.030 | 1.41 | 0.180 | 1.44 | 0.111 |
| rpmC | 50S ribosomal protein L29 | translation | 2.47 | 0.000 | 1.58 | 0.056 | 1.49 | 0.121 |
| rplJ | 50S ribosomal protein L10 | ribosome biogenesis, translation | 1.66 | 0.053 | 1.38 | 0.020 | 2.09 | 0.020 |

## Slide 5
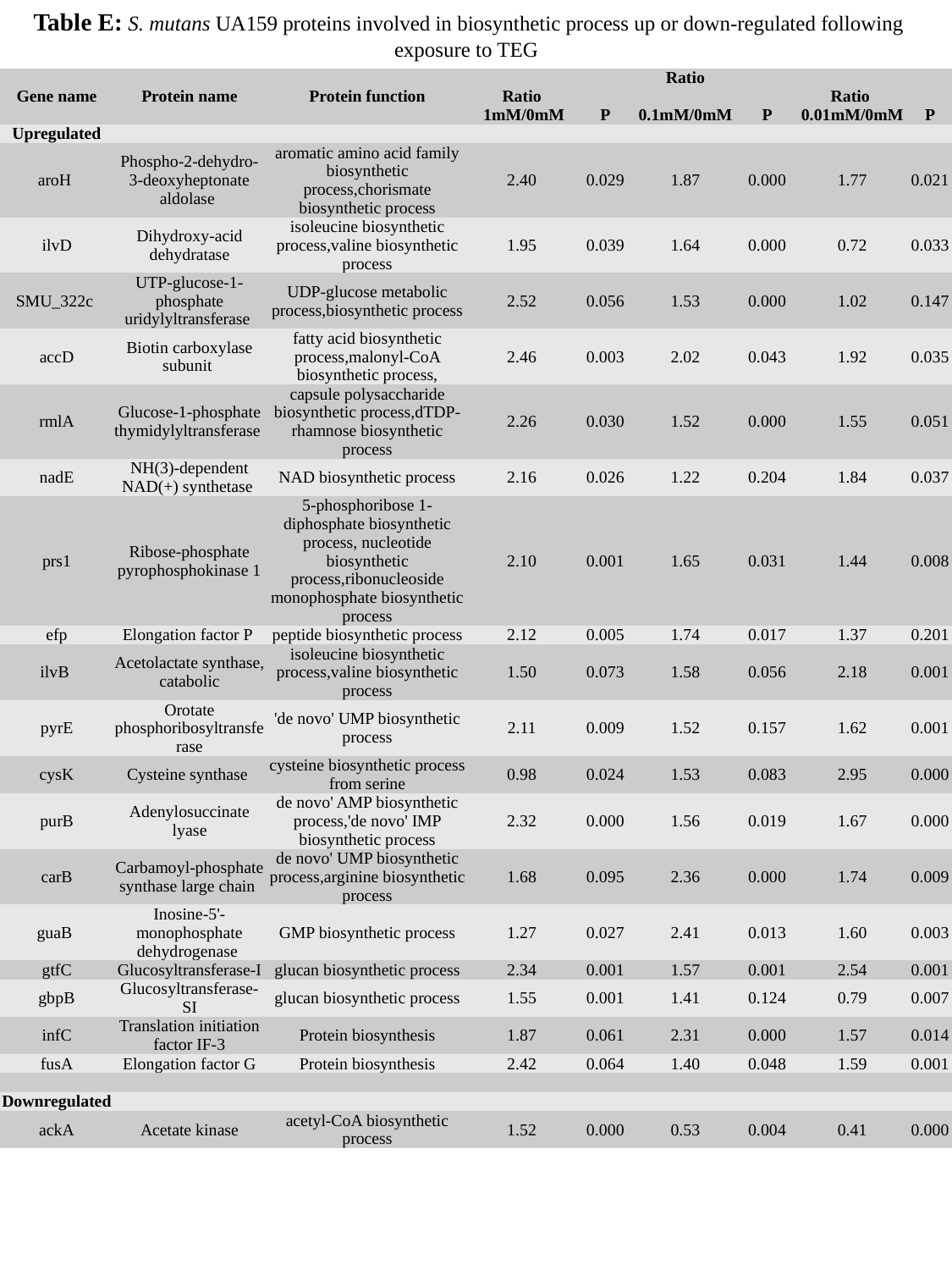

Table E: S. mutans UA159 proteins involved in biosynthetic process up or down-regulated following exposure to TEG
| Gene name | Protein name | Protein function | Ratio 1mM/0mM | P | Ratio 0.1mM/0mM | P | Ratio 0.01mM/0mM | P |
| --- | --- | --- | --- | --- | --- | --- | --- | --- |
| Upregulated | | | | | | | | |
| aroH | Phospho-2-dehydro-3-deoxyheptonate aldolase | aromatic amino acid family biosynthetic process,chorismate biosynthetic process | 2.40 | 0.029 | 1.87 | 0.000 | 1.77 | 0.021 |
| ilvD | Dihydroxy-acid dehydratase | isoleucine biosynthetic process,valine biosynthetic process | 1.95 | 0.039 | 1.64 | 0.000 | 0.72 | 0.033 |
| SMU\_322c | UTP-glucose-1-phosphate uridylyltransferase | UDP-glucose metabolic process,biosynthetic process | 2.52 | 0.056 | 1.53 | 0.000 | 1.02 | 0.147 |
| accD | Biotin carboxylase subunit | fatty acid biosynthetic process,malonyl-CoA biosynthetic process, | 2.46 | 0.003 | 2.02 | 0.043 | 1.92 | 0.035 |
| rmlA | Glucose-1-phosphate thymidylyltransferase | capsule polysaccharide biosynthetic process,dTDP-rhamnose biosynthetic process | 2.26 | 0.030 | 1.52 | 0.000 | 1.55 | 0.051 |
| nadE | NH(3)-dependent NAD(+) synthetase | NAD biosynthetic process | 2.16 | 0.026 | 1.22 | 0.204 | 1.84 | 0.037 |
| prs1 | Ribose-phosphate pyrophosphokinase 1 | 5-phosphoribose 1-diphosphate biosynthetic process, nucleotide biosynthetic process,ribonucleoside monophosphate biosynthetic process | 2.10 | 0.001 | 1.65 | 0.031 | 1.44 | 0.008 |
| efp | Elongation factor P | peptide biosynthetic process | 2.12 | 0.005 | 1.74 | 0.017 | 1.37 | 0.201 |
| ilvB | Acetolactate synthase, catabolic | isoleucine biosynthetic process,valine biosynthetic process | 1.50 | 0.073 | 1.58 | 0.056 | 2.18 | 0.001 |
| pyrE | Orotate phosphoribosyltransferase | 'de novo' UMP biosynthetic process | 2.11 | 0.009 | 1.52 | 0.157 | 1.62 | 0.001 |
| cysK | Cysteine synthase | cysteine biosynthetic process from serine | 0.98 | 0.024 | 1.53 | 0.083 | 2.95 | 0.000 |
| purB | Adenylosuccinate lyase | de novo' AMP biosynthetic process,'de novo' IMP biosynthetic process | 2.32 | 0.000 | 1.56 | 0.019 | 1.67 | 0.000 |
| carB | Carbamoyl-phosphate synthase large chain | de novo' UMP biosynthetic process,arginine biosynthetic process | 1.68 | 0.095 | 2.36 | 0.000 | 1.74 | 0.009 |
| guaB | Inosine-5'-monophosphate dehydrogenase | GMP biosynthetic process | 1.27 | 0.027 | 2.41 | 0.013 | 1.60 | 0.003 |
| gtfC | Glucosyltransferase-I | glucan biosynthetic process | 2.34 | 0.001 | 1.57 | 0.001 | 2.54 | 0.001 |
| gbpB | Glucosyltransferase-SI | glucan biosynthetic process | 1.55 | 0.001 | 1.41 | 0.124 | 0.79 | 0.007 |
| infC | Translation initiation factor IF-3 | Protein biosynthesis | 1.87 | 0.061 | 2.31 | 0.000 | 1.57 | 0.014 |
| fusA | Elongation factor G | Protein biosynthesis | 2.42 | 0.064 | 1.40 | 0.048 | 1.59 | 0.001 |
| | | | | | | | | |
| Downregulated | | | | | | | | |
| ackA | Acetate kinase | acetyl-CoA biosynthetic process | 1.52 | 0.000 | 0.53 | 0.004 | 0.41 | 0.000 |

## Slide 6
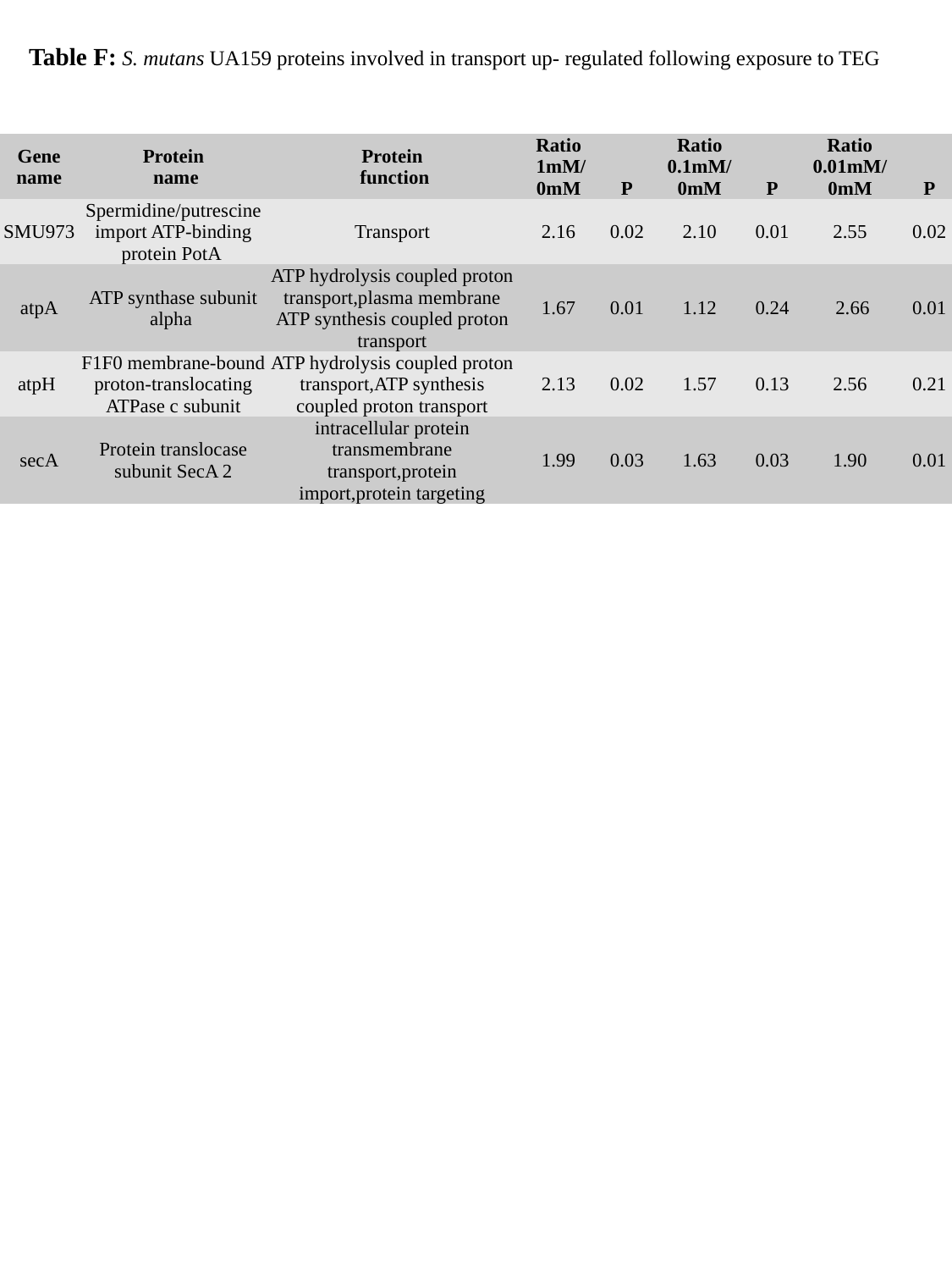

Table F: S. mutans UA159 proteins involved in transport up- regulated following exposure to TEG
| Gene name | Protein name | Protein function | Ratio 1mM/ 0mM | P | Ratio 0.1mM/ 0mM | P | Ratio 0.01mM/ 0mM | P |
| --- | --- | --- | --- | --- | --- | --- | --- | --- |
| SMU973 | Spermidine/putrescine import ATP-binding protein PotA | Transport | 2.16 | 0.02 | 2.10 | 0.01 | 2.55 | 0.02 |
| atpA | ATP synthase subunit alpha | ATP hydrolysis coupled proton transport,plasma membrane ATP synthesis coupled proton transport | 1.67 | 0.01 | 1.12 | 0.24 | 2.66 | 0.01 |
| atpH | F1F0 membrane-bound proton-translocating ATPase c subunit | ATP hydrolysis coupled proton transport,ATP synthesis coupled proton transport | 2.13 | 0.02 | 1.57 | 0.13 | 2.56 | 0.21 |
| secA | Protein translocase subunit SecA 2 | intracellular protein transmembrane transport,protein import,protein targeting | 1.99 | 0.03 | 1.63 | 0.03 | 1.90 | 0.01 |

## Slide 7
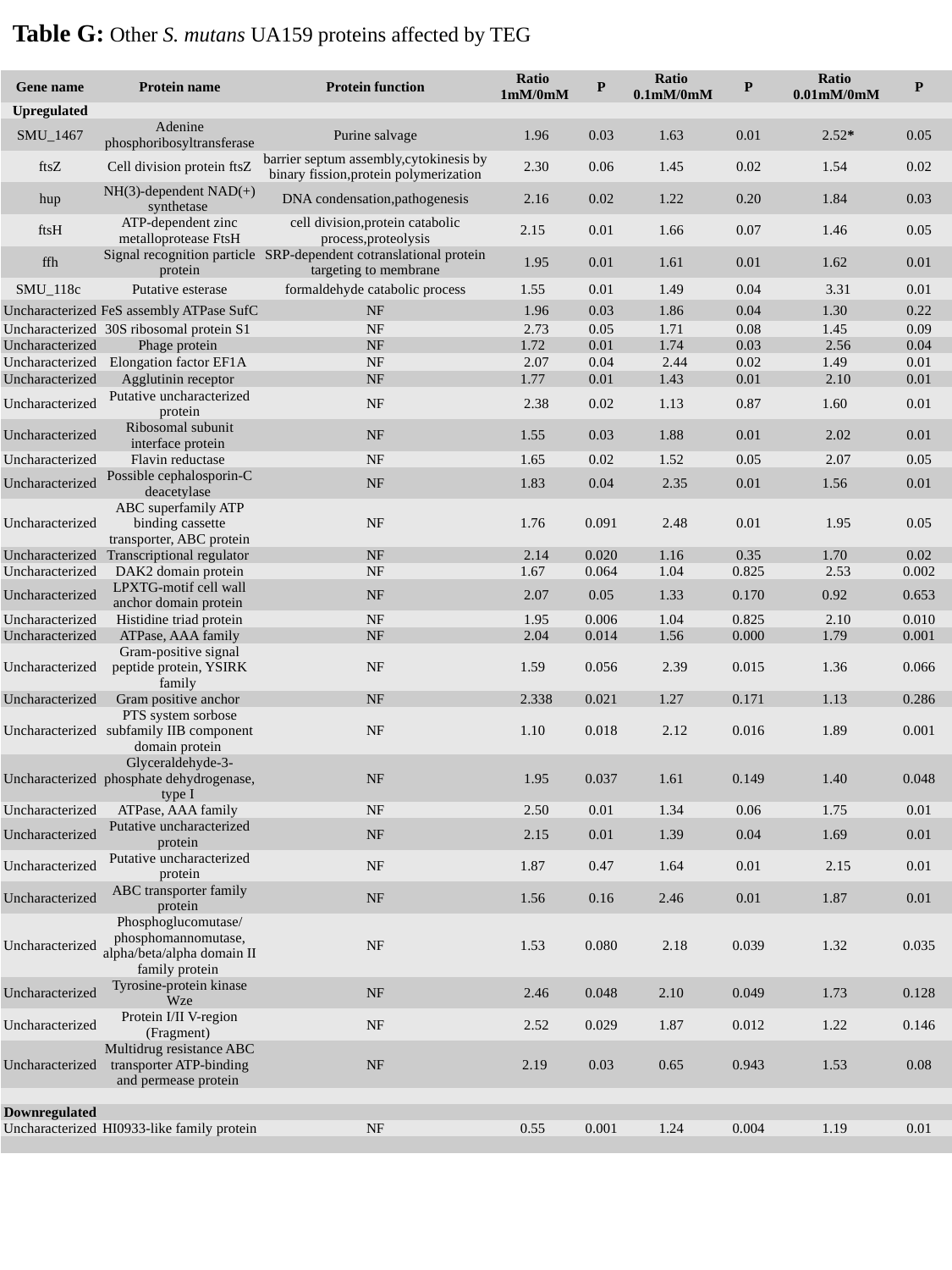

Table G: Other S. mutans UA159 proteins affected by TEG
| Gene name | Protein name | Protein function | Ratio 1mM/0mM | P | Ratio 0.1mM/0mM | P | Ratio 0.01mM/0mM | P |
| --- | --- | --- | --- | --- | --- | --- | --- | --- |
| Upregulated | | | | | | | | |
| SMU\_1467 | Adenine phosphoribosyltransferase | Purine salvage | 1.96 | 0.03 | 1.63 | 0.01 | 2.52\* | 0.05 |
| ftsZ | Cell division protein ftsZ | barrier septum assembly,cytokinesis by binary fission,protein polymerization | 2.30 | 0.06 | 1.45 | 0.02 | 1.54 | 0.02 |
| hup | NH(3)-dependent NAD(+) synthetase | DNA condensation,pathogenesis | 2.16 | 0.02 | 1.22 | 0.20 | 1.84 | 0.03 |
| ftsH | ATP-dependent zinc metalloprotease FtsH | cell division,protein catabolic process,proteolysis | 2.15 | 0.01 | 1.66 | 0.07 | 1.46 | 0.05 |
| ffh | Signal recognition particle protein | SRP-dependent cotranslational protein targeting to membrane | 1.95 | 0.01 | 1.61 | 0.01 | 1.62 | 0.01 |
| SMU\_118c | Putative esterase | formaldehyde catabolic process | 1.55 | 0.01 | 1.49 | 0.04 | 3.31 | 0.01 |
| Uncharacterized | FeS assembly ATPase SufC | NF | 1.96 | 0.03 | 1.86 | 0.04 | 1.30 | 0.22 |
| Uncharacterized | 30S ribosomal protein S1 | NF | 2.73 | 0.05 | 1.71 | 0.08 | 1.45 | 0.09 |
| Uncharacterized | Phage protein | NF | 1.72 | 0.01 | 1.74 | 0.03 | 2.56 | 0.04 |
| Uncharacterized | Elongation factor EF1A | NF | 2.07 | 0.04 | 2.44 | 0.02 | 1.49 | 0.01 |
| Uncharacterized | Agglutinin receptor | NF | 1.77 | 0.01 | 1.43 | 0.01 | 2.10 | 0.01 |
| Uncharacterized | Putative uncharacterized protein | NF | 2.38 | 0.02 | 1.13 | 0.87 | 1.60 | 0.01 |
| Uncharacterized | Ribosomal subunit interface protein | NF | 1.55 | 0.03 | 1.88 | 0.01 | 2.02 | 0.01 |
| Uncharacterized | Flavin reductase | NF | 1.65 | 0.02 | 1.52 | 0.05 | 2.07 | 0.05 |
| Uncharacterized | Possible cephalosporin-C deacetylase | NF | 1.83 | 0.04 | 2.35 | 0.01 | 1.56 | 0.01 |
| Uncharacterized | ABC superfamily ATP binding cassette transporter, ABC protein | NF | 1.76 | 0.091 | 2.48 | 0.01 | 1.95 | 0.05 |
| Uncharacterized | Transcriptional regulator | NF | 2.14 | 0.020 | 1.16 | 0.35 | 1.70 | 0.02 |
| Uncharacterized | DAK2 domain protein | NF | 1.67 | 0.064 | 1.04 | 0.825 | 2.53 | 0.002 |
| Uncharacterized | LPXTG-motif cell wall anchor domain protein | NF | 2.07 | 0.05 | 1.33 | 0.170 | 0.92 | 0.653 |
| Uncharacterized | Histidine triad protein | NF | 1.95 | 0.006 | 1.04 | 0.825 | 2.10 | 0.010 |
| Uncharacterized | ATPase, AAA family | NF | 2.04 | 0.014 | 1.56 | 0.000 | 1.79 | 0.001 |
| Uncharacterized | Gram-positive signal peptide protein, YSIRK family | NF | 1.59 | 0.056 | 2.39 | 0.015 | 1.36 | 0.066 |
| Uncharacterized | Gram positive anchor | NF | 2.338 | 0.021 | 1.27 | 0.171 | 1.13 | 0.286 |
| Uncharacterized | PTS system sorbose subfamily IIB component domain protein | NF | 1.10 | 0.018 | 2.12 | 0.016 | 1.89 | 0.001 |
| Uncharacterized | Glyceraldehyde-3-phosphate dehydrogenase, type I | NF | 1.95 | 0.037 | 1.61 | 0.149 | 1.40 | 0.048 |
| Uncharacterized | ATPase, AAA family | NF | 2.50 | 0.01 | 1.34 | 0.06 | 1.75 | 0.01 |
| Uncharacterized | Putative uncharacterized protein | NF | 2.15 | 0.01 | 1.39 | 0.04 | 1.69 | 0.01 |
| Uncharacterized | Putative uncharacterized protein | NF | 1.87 | 0.47 | 1.64 | 0.01 | 2.15 | 0.01 |
| Uncharacterized | ABC transporter family protein | NF | 1.56 | 0.16 | 2.46 | 0.01 | 1.87 | 0.01 |
| Uncharacterized | Phosphoglucomutase/phosphomannomutase, alpha/beta/alpha domain II family protein | NF | 1.53 | 0.080 | 2.18 | 0.039 | 1.32 | 0.035 |
| Uncharacterized | Tyrosine-protein kinase Wze | NF | 2.46 | 0.048 | 2.10 | 0.049 | 1.73 | 0.128 |
| Uncharacterized | Protein I/II V-region (Fragment) | NF | 2.52 | 0.029 | 1.87 | 0.012 | 1.22 | 0.146 |
| Uncharacterized | Multidrug resistance ABC transporter ATP-binding and permease protein | NF | 2.19 | 0.03 | 0.65 | 0.943 | 1.53 | 0.08 |
| | | | | | | | | |
| Downregulated | | | | | | | | |
| Uncharacterized | HI0933-like family protein | NF | 0.55 | 0.001 | 1.24 | 0.004 | 1.19 | 0.01 |
| | | | | | | | | |

## Slide 8
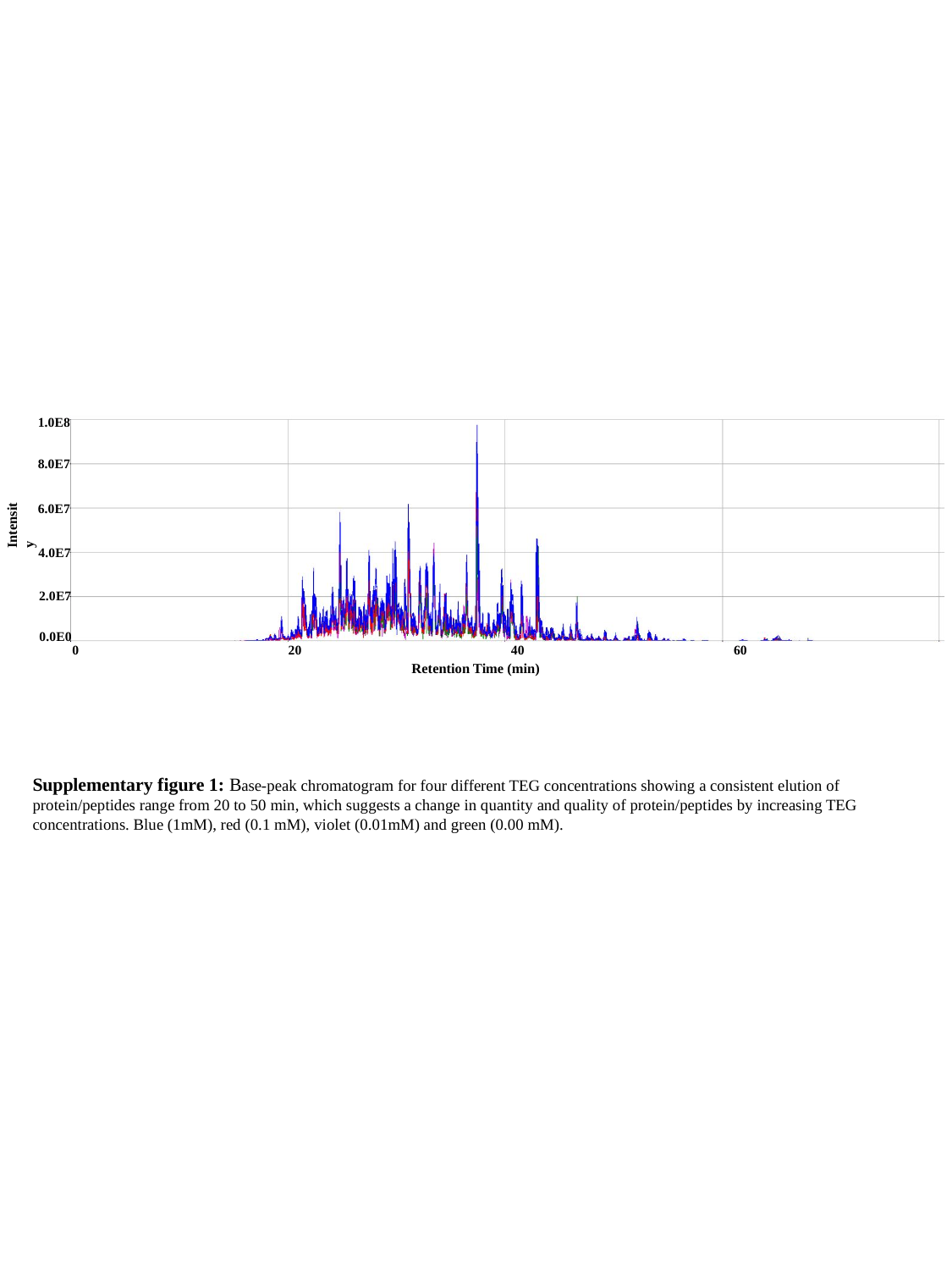

1.0E8
8.0E7
6.0E7
Intensity
4.0E7
2.0E7
0.0E0
0 20 40 60 80
Retention Time (min)
Supplementary figure 1: Base-peak chromatogram for four different TEG concentrations showing a consistent elution of protein/peptides range from 20 to 50 min, which suggests a change in quantity and quality of protein/peptides by increasing TEG concentrations. Blue (1mM), red (0.1 mM), violet (0.01mM) and green (0.00 mM).
